# Molecular Diversity of Lupane Hybrids in Drug Design and Materials Science

**DOI:** 10.3390/molecules30204108

**Published:** 2025-10-16

**Authors:** Victoria V. Lipson, Maria G. Shirobokova, Mustafa Kemal Gümüş, Arda Ozturkcan, Valentyn A. Chebanov

**Affiliations:** 1 Institute of Functional Materials Chemistry, State Scientific Institution “Institute for Single Crystals” of NAS of Ukraine, 60 Nauky Ave., 61072 Kharkiv, Ukraine; 2 Chemistry School, V.N. Karazin Kharkiv National University, 4 Svobody Sq., 61022 Kharkiv, Ukraine; 3 Science-Technology Research and Application Center, Artvin Coruh University, Seyitler Campus, Artvin 08100, Türkiye; 4 Department of Nutrition and Dietetics, Faculty of Health Sciences, Istanbul Gelisim University, Istanbul 34310, Türkiye; sozturkcan@gelisim.edu.tr

**Keywords:** lupane triterpenoids, betulinic acid, biological activity, liquid crystals, chiral dopants, organogels

## Abstract

The need for new, more effective drugs to treat cancer, infectious diseases, various parasitic infestations, and metabolic disorders requires innovative approaches to the design of promising molecules. One of these areas is the creation of hybrid structures. Lupane triterpenoids are of significant interest for such research due to their high abundance in natural sources and their renewable nature, their molecular architecture, presence of several easily modifiable functional groups, enantiomeric purity, broad spectrum of biological activity, and low toxicity. Active research into the biological properties of new pentacyclic triterpenoid derivatives, not only of the lupane series but also of the oleonane and ursane series, is evidenced by the large number of reviews and experimental studies devoted to this topic. Our interest in the modification of lupanoids stems not only from the search for biologically active compounds but also from the development of functional materials. However, the materials science aspects of lupanoid applications are virtually unknown in literature. We have tried to fill this gap and examined the possibility of using betulin derivatives to create advanced materials. The high lipophilicity and nanoscale molecular structure of these compounds make them highly promising as chiral dopants in liquid crystal compositions and organogel components.

## 1. Introduction

**Figure 1:**
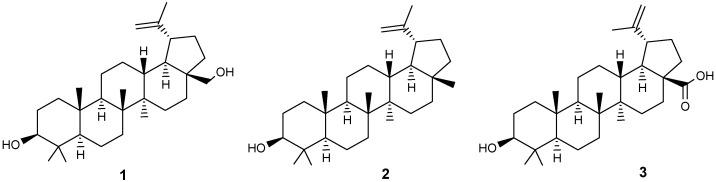
The most common triterpenoids of the lupane series.

Triterpenoids of the lupane series, such as betulin (BE) **1**, lupeol **2** and betulinic acid (BA) **3** (Figure 1), have been attractive molecular platforms for the synthesis of biologically active hybrid compounds, conjugates or so-called chimeras in the last two decades, in the structure of which, in addition to the indicated pentacyclic fragments, both natural substituents with known activity and synthetic ones may be present.

The interest in these plant metabolites is due to their biological activity [1,2,3,4,5,6], the wide distribution of natural sources and their renewability, enantiomeric purity, the presence of easily modifiable functional groups in their structure, and low toxicity. Lupanoids are constituents of certain foods and are found in various Mediterranean spices and fruits. For instance, BA can be detected in olive oil and in the leaves of *Rosmarinus officinalis* [7,8,9]. The primary source of betulin (BE) **1** is the bark of trees, considered industrially significant. The outer layer of birch bark contains 10–35% BE, depending on the birch species, growth location and conditions, tree age, and other factors [10]. Lupeol **2** constitutes about 10%, while betulinic acid (BA) **3** accounts for less than 2% of the BE content in *Betula alba* bark. The bark of *Platanus acerifolia* contains approximately 3% BA **3** by dry weight and serves as a higher-yielding source [9]. Various extraction methods, including stirring extraction, Soxhlet extraction, ultrasonic extraction, and microwave-assisted extraction, are employed to isolate this acid from natural materials [10]. BA can be synthesized from BE through a five-step process with yields reaching up to 50% [11]. Biotechnological production methods for BA have also been established, utilizing *Saccharomyces cerevisiae* [12].

**Figure 2:**
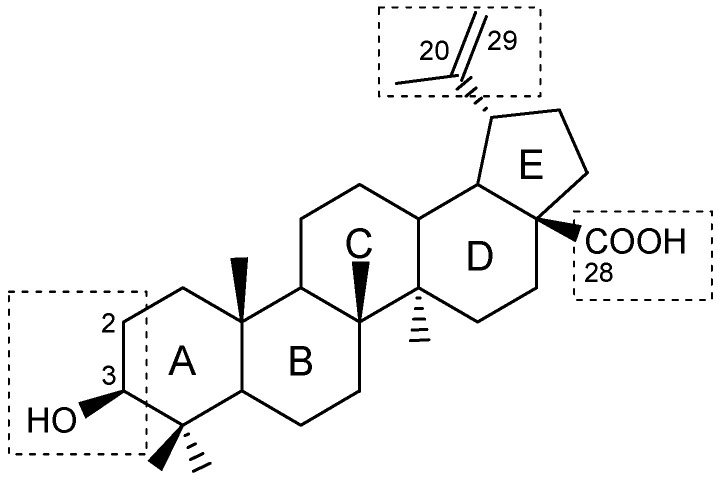
The positions in the lupane molecular platform amenable to chemical modification.

There are two primary methods for constructing lupane hybrids. One approach introduces into a known compound of natural origin a fragment of a natural molecule that facilitates targeted delivery, or a structural component with antioxidant properties that may reduce toxicity and affect the intended outcome [13]. Another method involves covalently combining natural and synthetic compounds to modify the pharmacodynamic properties of both molecules. Compounds that form conjugates must act on at least two pathogenetically related links of a disease, and the hybrid should be designed in such a way as to cause specific effects in both directions. Many natural products with various pharmacological properties are significantly inferior in activity or selectivity to those of synthetic biologically active substances. The development of hybrids based on lupanoids represents one approach to enhance specific activity in targeted directions. Currently, methods have been developed for modifying the lupane molecular platform (Figure 2) in positions C-2, C-3, C-28, the isopropenyl fragment, and through the opening of ring A [2,4,14].

In this case, both condensed systems and those connected by linkers can be synthesized. The latter are more conformationally flexible compounds, which facilitates their access to the active sites of target proteins. Additionally, fragments that enhance water solubility of the target products can be incorporated into the linkers. An increase in the area of the triterpenoid molecular platform through the annulation of additional rings may result in a notable reduction in the solubility of these already sparingly soluble substances. In this regard, it is advisable that the introduction of additional cycles is accompanied by the appearance of fragments in the molecules that are capable of ionization or the formation of hydrogen bonds. This will have a positive effect on water solubility and lead to an increase in potential binding sites with biological targets, which in turn will increase the activity of such conjugates.

The development of hybrid compounds based on the pentacyclic triterpenoid scaffold for pharmaceutical purposes has been widely covered in several reviews [1,2,3,4,5,6]. Some of them focus on methods of chemical modification of pentacyclic triterpenoids not only of the lupane, but also of the ursane and oleanane types [4,6]. Publications dealing with the biological properties of triterpenoid derivatives mainly concern their antitumor and antiviral activity [1,5,15,16,17,18,19]. Thus, in a recently published review by Wang J. et al., only the anticancer properties of lupanoids were discussed [3]. The prospects for using hybrid compounds based on BE and BA in the development of anti-inflammatory, antibacterial, antiparasitic agents and drugs for the correction of metabolic disorders much less addressed in the literature. Information on the use of lupanoids in materials science is extremely limited and is not presented in any review. In our review, we examine the molecular diversity of lupane hybrids with regard to their role as biologically active compounds with different modes of action, and as components of liquid crystal materials and organic gelling agents.

## 2. Antitumor Properties of Lupanoid Derivatives

The anticancer activity of lupane derivatives, particularly various esters and amides of BA, represents the most frequently examined area in current literature. However, despite this, for the sake of completeness of the analysis of the pharmacologically significant properties of lupanoids, we decided not to ignore this type of activity from consideration. Many of these compounds are conjugates with established antitumor agents or naturally occurring products [3,5,6,8,15,16,17,18,19,20,21,22,23,24,25,26,27,28,29,30,31,32,33,34,35,36,37,38,39,40,41,42,43,44,45,46,47,48,49,50]. Interest in studying BA hybrids arose from the discovery of this acid’s ability to induce apoptosis in melanoma cell lines MEL 1, 2, 3, and 4, where EC_50_ values ranged from 0.5 to 4.8 μg/mL. Notably, BA had no toxic effect on normal cells at concentrations up to 100 μg/kg body weight [20,21]. Subsequent studies demonstrated that this acid inhibits the epithelial-to-mesenchymal transition process in melanoma, which is implicated in both metastatic spread and resistance to cancer therapies [22]. At present, BA is recognised for its anticancer activity, with an IC_50_ concentration range of 2.5–12.99 μg/mL observed against a variety of solid tumour cell lines, including cervical cancer (HeLa), breast cancer (MDA MB 231), lung carcinoma (A549), colorectal carcinoma, gastric adenocarcinoma (AGS), prostate cancer, bladder cancer (253JB-V and KU-7), and endometrial adenocarcinoma [1,23]. Notably, BA exhibits significant anti-proliferative and pro-apoptotic effects in human multiple myeloma KM3 cells within a dose range of 5–25 μg/mL [24]. Across all cell lines evaluated in the referenced study [25]—such as SKW6, HUT 78, CEM T cell leukemia; BJAB, NALM6, BOE B cell; and HL 60 myeloid cell line—samples displayed sensitivity to BA-induced apoptosis at a concentration of 10 μg/mL.

### 2.1. BA-rhodamine and BA-triphenylphosphonium Conjugates as Mitochondrial Disruptors

**Figure 3:**
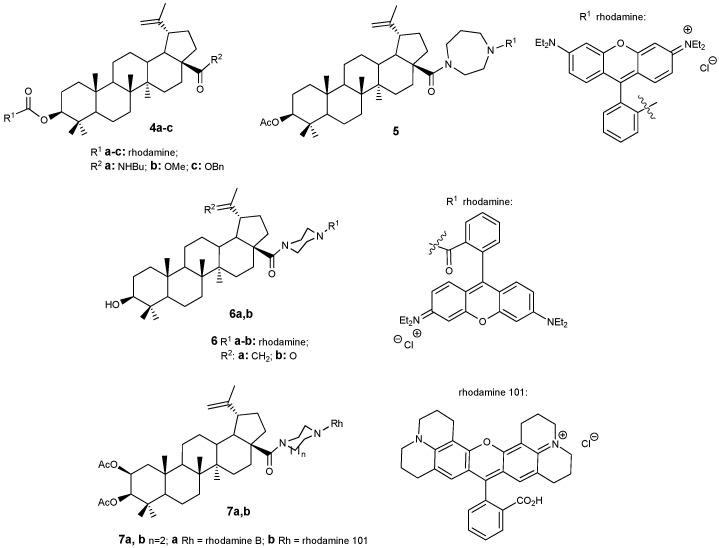
BA-rhodamine hybrids with antitumor activity.

The cytotoxic effects of lupanoids are primarily linked to their capacity to disrupt mitochondrial function in cancer cells, leading to apoptosis. Therefore, integrating the lupane molecular scaffold with compounds that preferentially accumulate within mitochondria is recommended. Rhodamine and triphenylphosphonium salts exhibit such organelle-targeting properties. Additionally, the intrinsic fluorescence of rhodamine enables the application of these conjugates in diagnostic procedures or for investigating mechanisms of action. BA-rhodamine hybrids **4**–**6** (Figure 3) were synthesized and evaluated against seven human cancer cell lines [4]. Rhodamine B did not demonstrate cytotoxicity at concentrations up to 30 μM, whereas the triterpenoid rhodamine B hybrids containing a piperazine spacer (**6a**,**b**) exhibited activity with IC_50_ values ranging from 0.046 to 0.176 μM. Acetylation of the 2-hydroxy group also contributes to increased cytotoxic activity. However, studied compounds showed limited selectivity, as their IC_50_ values in cancer cell lines were like those observed in healthy mouse fibroblasts NIH 3T3 (IC_50_–0.208 μM) [26].

Recently, in continuation of these studies, the authors of work [27] obtained new conjugates of 2,3-diacetyloxybetulinic acid with rhodamines B and 101, in which a piperazine linker was also used to join the target fragments. Hybrids **7a**,**b** demonstrated the highest cytotoxic activity against A2780 tumor cells (IC_50_ = 0.016 and 0.019 μM, respectively). These compounds in contrast with their analogs **6** exhibited high selectivity for A2780 cells compared to normal fibroblasts NIH 3T3. Cell cycle studies on A375 melanoma cells showed that conjugates **7a**,**b** inhibit cell division in the G1/G0 phase. Using flow cytometry with Annexin V-FITC/PI staining, it was established that the effect of compounds **7a**,**b** causes both necrosis and apoptosis of tumor cells.

The presence of a rhodamine fragment with luminescent properties allows for tracking the movement and accumulation of the corresponding conjugates in organs and tissues in in vivo experiments. In the future, such hybrids may find application in theranostics.

**Figure 4:**
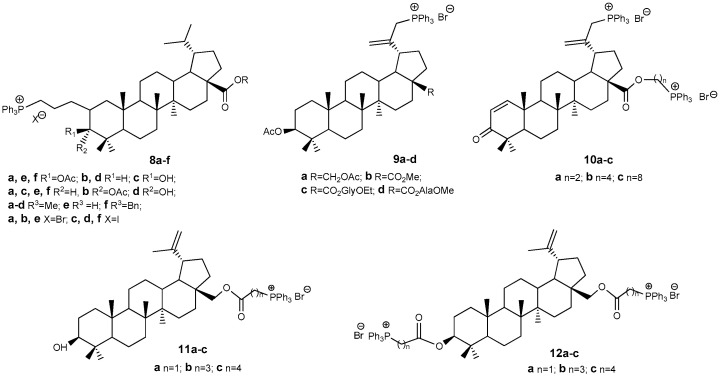
BA-triphenylphosphonium conjugates with antitumor properties.

Triphenylphosphonium derivatives of BE and BA induce tumor cell apoptosis via mitochondrial damage, due to their positively charged, lipophilic Ph_3_P^+^ fragment. Unlike rhodamine, these conjugates are less toxic to normal cells [28,29,30]. Modifying the lupane platform in this way has proven highly effective. Since the initial report in 2013 [28] detailing the synthesis of triphenylphosphonium conjugates **8a**–**f** (Figure 4), derived from BA esters, over 730 publications have explored the synthesis and antitumor characteristics of compounds bearing Ph_3_P^+^ substituents linked to the lupane core structure either directly or via various linkers at positions C-3, C-28, and C-30. The hybrids **8a**–**f** exhibited cytotoxic activity against P-815 and Ehrlich tumor cells, with IC_50_ values ranging from 1.1 to 4.7 μM; in comparison, BA demonstrated IC_50_ values of 41 and 54 μM for these cell lines, respectively. Additionally, subsequent work by the same authors [29] described compounds **9a**–**d, 10a**–**c**, which displayed cytotoxicity approximately ten times greater than that observed with BA.

Triphenylphosphonium derivatives of BE **11a**–**c** and **12a**–**c** were assessed for their cytotoxicity against various cell lines, including prostate adenocarcinoma PC-3 (IC_50_: 6.9–0.12 μM), human breast carcinoma MCF-7 (IC_50_: 8.7–0.43 μM), vinblastine-resistant human breast cancer MCF-7/Vinb (IC_50_: 10–0.045 μM), and human skin fibroblast HSF (IC_50_: 9.9–0.76 μM). By comparison, the IC_50_ values of BE ranged from 233.4 to 148.3 μM across the same cell types. Structure-activity correlation analysis indicated that the cytotoxicity of betulin conjugates with Ph_3_P^+^ is significantly greater than that of native BE, with activity increasing in the following order: **11a** ~ **12a** < **11c** ~ **12b** ~ **12c** < **11b**. Notably, the inclusion of a methylene linker does not enhance biological activity, whereas propylene and butylene linkers confer increased efficacy [5,30].

### 2.2. BA-cisplatin Complexes

**Figure 5:**
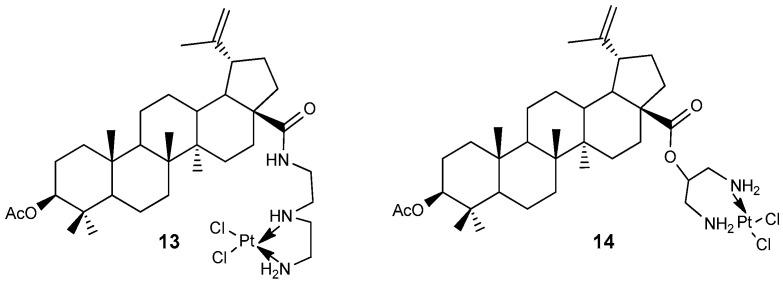
BA-cisplatin complexes.

A series of lupanoid conjugates were synthesized to enhance their potential anticancer activity by incorporating another established molecule, such as cisplatin, with the aim of achieving greater efficacy, improved selectivity, or reduced toxicity compared to the parent compounds. For instance, BA-cisplatin complexes **13** and **14** (Figure 5) were evaluated for their anti-proliferative effects against tumor cell lines A549, A2780, 8505C, 518A2, and MCF-7. These complexes demonstrated activity with IC_50_ values of 11.57–17.32 µM for **13** and 13.13–29.83 µM for **14**, which were comparable to those of the original BA compound (IC_50_ = 8.75–14.8 µM). Notably, these conjugates displayed lower cytotoxicity than the reference drug, cisplatin (IC_50_ = 0.33–1.34 µM), across all tested tumor cell lines [31].

### 2.3. Lupanoid Conjugates with Natural Products and Their Antitumor Activity

**Figure 6:**
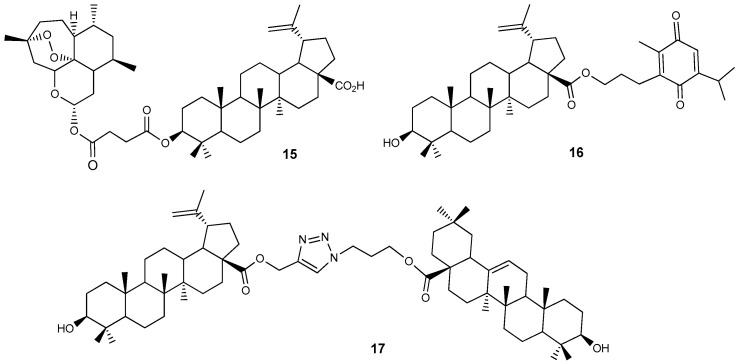
BA hybrids with plant metabolites.

Hybrid compounds that combine two natural components within a single molecular system have been studied as possible cytotoxic agents. Artemisinin, a metabolite from *Artemisia annua*, is recognized as an antimalarial compound, while thymoquinone is found in the essential oil of *Nigella sativa*. Their conjugates **15** and **16** with BA (Figure 6) were synthesized and evaluated for cytotoxic effects on glioma cancer cells, neurons, and astrocytes. At a dose of 10 µM, these compounds demonstrated selective cytotoxicity towards glioma cells and inhibited their migration [32].

Pattnaik et al. found that their synthesized BA-ursolic acid triazole dimer **16** (Figure 6) had a cytostatic effect against MCF-7 and MDA-MB-231 breast cancer cells that was 2.9 times greater (GI_50_ = 1.4 μM) than doxorubicin (GI_50_ = 4.1 μM) for MDA-MB-231 cells [33].

**Figure 7:**
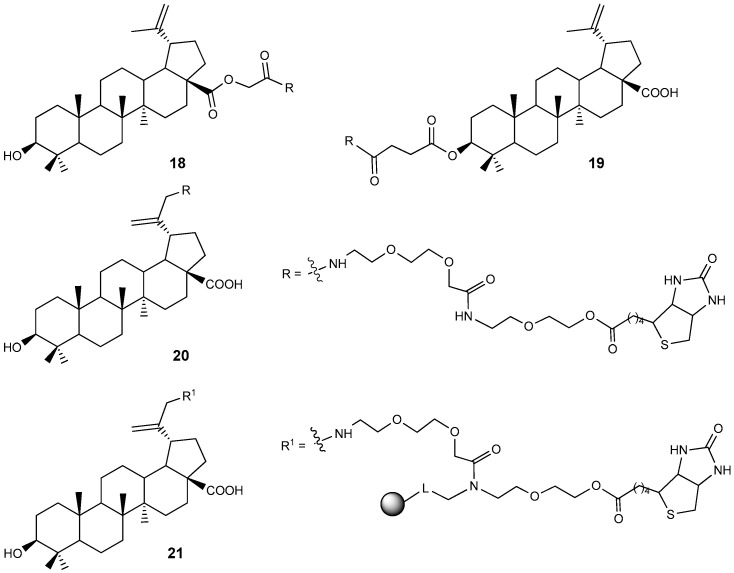
BA-biotin PEG linked conjugates.

Various BA conjugates with biotin attached at the 3-C, C-28, and C-30 positions via a PEG linker have been synthesized [34]. The cytotoxicity of compounds **18**–**21** (Figure 7) was evaluated on CCRF-CEM and HCT116 tumor cell lines. Conjugate **18** exhibited an IC_50_ value of 14.85 µM, which is approximately twice as active as BA **1** (IC_50_ = 30.0 µM) against human leukemic lymphoblasts (CCRF-CEM cells).

**Figure 8:**
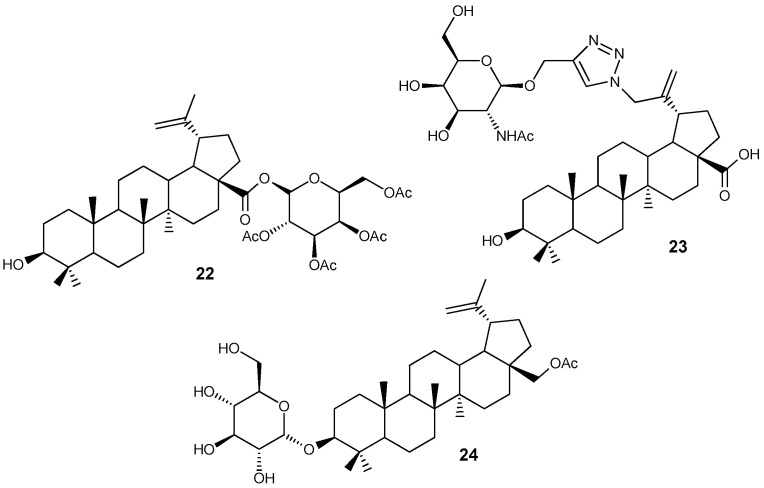
BA and BE sugar hybrids.

Interest in modifying natural compounds with fragments of sugars and amino sugars is due to the active entry of the latter into cancer cells, and therefore they can be used as carriers of potential antineoplastic agents into target cells. The articles [35,36,37] describe β-D-glucopyranosyl and N-acetyl-β-D-galactopyranosylamine conjugates BA **22**, **23** and BE **24** (Figure 8), in the molecules of which the carbohydrate fragment is linked to the lupane platform by an ether bond or via a 1,2,3-triazole spacer. Hybrid **22** showed activity against CEM, MCF-7, HeLa and G361 tumor cell lines (IC_50_ 2.8–8.6 μM) [35], and compound **24** suppressed the growth of U251MG, U343MG and LN229 cells (IC_50_ 8.1–17.2 μM) and reduced the expression of the intracellular apoptosis inhibitor Survivin [36].It was noted that complete acetylation of the sugar fragment in promoted an increase in the activity of **22**, while the introduction of a 1,2,3-triazole ring into compound **23**, on the contrary, reduced the effect (IC_50_ > 100 μM) [37].

### 2.4. BA Analogues as Topoisomerase I and IIA Inhibitors

**Figure 9:**
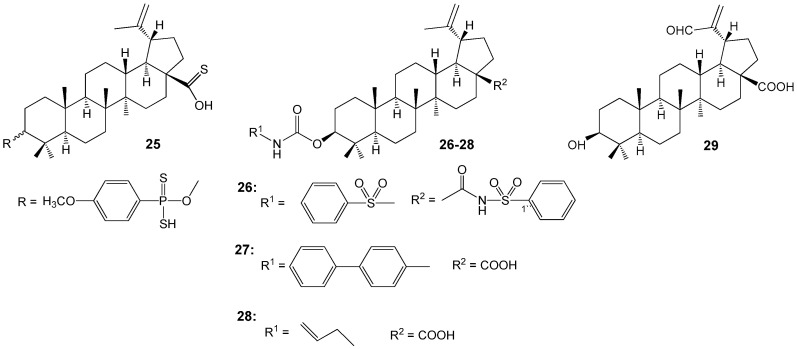
BA analogous with topoisomerase I and IIA inhibitory activity.

Abdel Bar FM et al. synthesized several BA analogues (Figure 9) and evaluated their inhibitory activity on topoisomerase I and IIA. Compounds **25**, **26**, and **29** exhibited topoisomerase I inhibitory activities like or exceeding those of camptothecin, a known anticancer drug. Additionally, BA derivatives **25**–**28** demonstrated approximately 1.5 times higher activity than etoposide in topoisomerase IIA assays and showed increased cytotoxicity against human colon cancer cell lines SW948 and HCT-116 as well as the breast cancer cell line MDA-MB-231, when compared with the parent acid. Structure-activity relationship analysis indicated that aromatic or olefinic substitutions at the C-3 position of the lupane scaffold significantly enhanced binding affinity for both topoisomerase types. In contrast, oxidation of the isopropenyl side chain to an aldehyde **29** produced moderate inhibitory activity against topoisomerase I, without substantially affecting the binding affinity for topoisomerase IIA [38].

### 2.5. BA and BE Sulfomate and Sulfonamides with Antitumor and Carbonic Anhydrase Inhibition Activity

**Figure 10:**
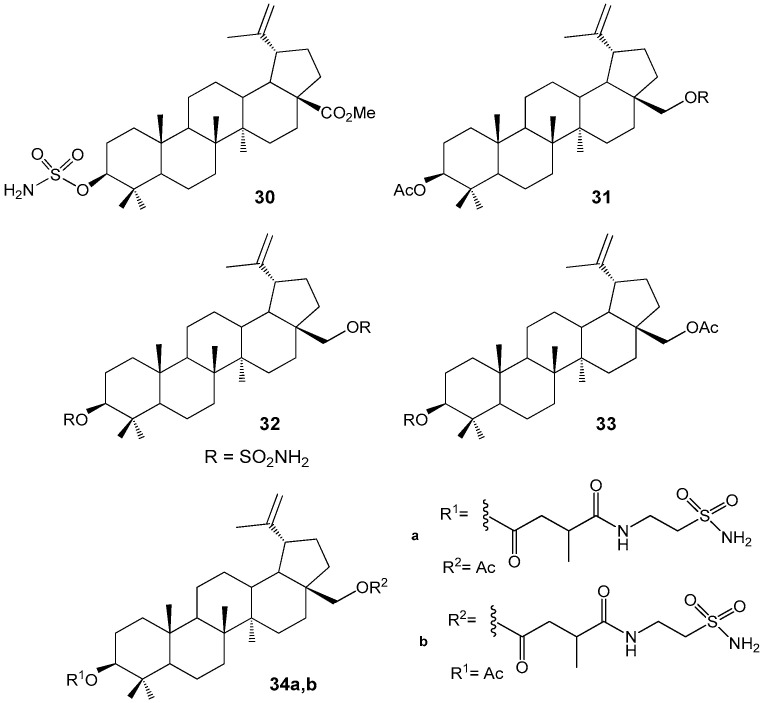
Sulfamate and sulfonamides derivatives of BA and BE with cytotoxic activity.

Sulfamate derivatives of BA **30**–**33** (Figure 10) were synthesized and evaluated for their antiproliferative activity against the tumor cell lines 518A2, FaDu, HT-29, MCF-7, A549, and SW1736. Compound **30** (EC_50_ = 6.7–10.1 µM) and the C-28-monosubstituted derivative **33** (IC_50_ = 4.87–9.94 µM) demonstrated significant cytotoxic effects on 518A2, 8505C, A2780, MCF-7, and A54 cell lines. Additionally, compound **33** exhibited potent inhibitory activity against carbonic anhydrase IX (Ki = 1.25 nM). The combination of antitumor activity with the inhibition of carbonic anhydrase improves the prognosis in breast cancer therapy [39,40]. Similar activity against several cell lines MCF-7, HS578T, MDA-MB-231, BT-20, T47D and SKBR of breast cancer (IC_50_ 8–14 µM) in combination with inhibition of carbonic anhydrase IX and XII was also observed with BE sulfonamides **34a**,**b**. These compounds are considered as promising lead structures for further pharmacological trials [41].

### 2.6. BA, BE and Lupeol Conjugates with Heterocycles

**Figure 11:**
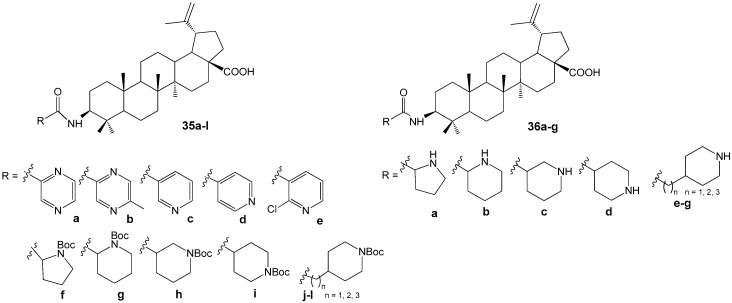
Conjugates of BA with nitrogen-containing heterocycles.

The literature provides extensive information on the synthesis and antitumor properties of conjugates formed from lupanoids and nitrogen-containing heterocycles. The heterocyclic fragment may be attached to the lupane structure via an amide or ester bond at functional groups located at the C-2, C-3, or C-28 positions, as well as through various linkers, or may form fused systems. Compound **35l** (Figure 11) demonstrated the highest activity against the proliferation of eight non-drug-resistant and one multidrug-resistant tumor cell lines among the hybrids with different heterocyclic substituents at the C-3 position. The cytotoxicity (IC_50_ = 0.33–2.45 µM) observed in the multidrug-resistant human breast cancer cell line (MCF-7/ADR) was approximately 20-fold greater than that of the parent compound BA (IC_50_ = 14.04–38.50 µM). Structure-activity relationship analysis within compound groups **35** and **36** indicated that conjugates containing a saturated heterocyclic fragment exhibited higher cytotoxicity compared to analogs featuring a heteroaromatic substituent. Furthermore, a reduction in antitumor activity was noted as the distance between the amide bond and the piperidine ring increased [42].

**Figure 12:**
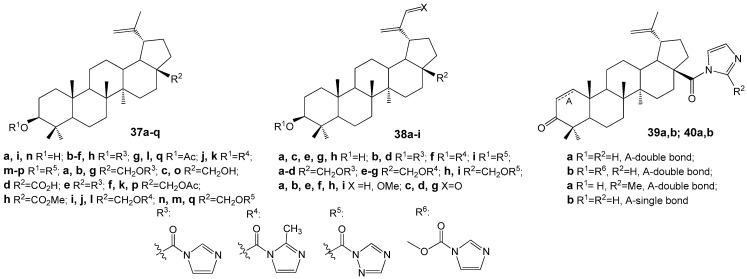
BE and BA imidazole, 2-methylimidazole or 1,2,4-triazole hybrids.

Conjugates of BE and BA with imidazole, 2-methylimidazole, or 1,2,4-triazole (Figure 12) were evaluated across several cancer cell lines. Most compounds demonstrated moderate to high cytotoxic activity, particularly in HepG2 (hepatocellular carcinoma), Jurkat (T-lymphocytic leukemia), and HeLa (cervical carcinoma) cell lines. The derivatives with the imidazole moiety R^3^ at position C-3 (**37c**, IC_50_ of 2.0 µM in HepG2) or C-28 (**39a**, IC_50_ of 1.7 µM in HepG2; **40b**, IC_50_ of 0.8 µM in HepG2) exhibited the lowest IC_50_ values [4,43].

**Figure 13:**
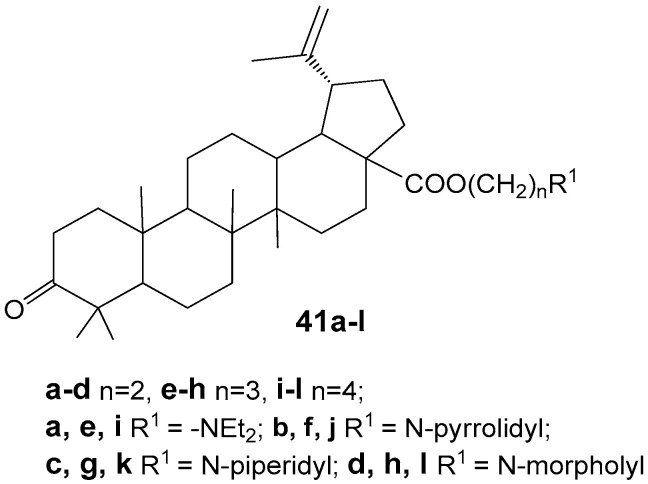
Betulonic acid conjugates with aliphatic and heterocyclic tertiary amines.

Yang S.-J. et al. reported the synthesis of betulonic acid derivatives **41a**–**l** and evaluated their in vitro anti-cancer activity against MGC-803, PC3, Bcap-37, A375, and MCF-7 cell lines [44]. The compound exhibiting the highest activity, **41k** (Figure 13), demonstrated IC_50_ values of 3.6, 5.6, 4.2, 7.8, and 5.2 µM on these respective cancer cell lines. Further studies indicated that this hybrid, **41k**, induces apoptosis in MGC-803 cells via the mitochondrial intrinsic pathway.

**Figure 14:**
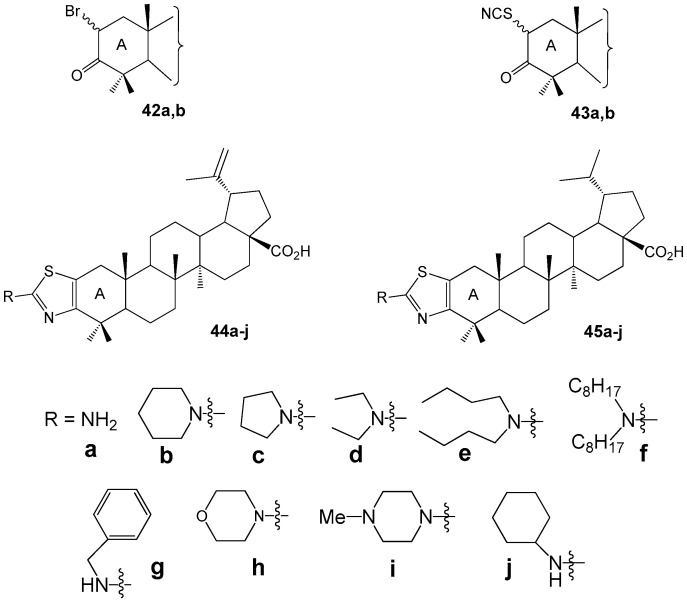
Betulonic and dihydrobetulonic acid derivatives with cytotoxic activity.

Borkova L. et al. synthesized thiazole-modified betulonic acids **44a**–**j** and dihydrobetulonic acids **45a**–**j** from 2-bromo-3-oxo triterpenoids **42a**,**b** through 2-thiocyanato-3-oxo derivatives **43a**,**b** (Figure 14). These compounds were evaluated for their in vitro cytotoxic effects across eight cancer cell lines [45]. Compound **44a** induced apoptosis in CCRF-CEM cells (acute lymphoblastic leukemia) via the internal pathway (IC_50_ 2.4 µM). The 2α/2β-bromo dihydrobetulonic acid **42b** demonstrated effectiveness against the K562 leukemic cell line (IC_50_ 0.7 µM), and its activity in the colon cancer HCT116 cell line was IC_50_ 1.0 µM. 2α/2β-Thiocyanato dihydrobetulonic acid **43b** showed cytotoxicity toward both normal K562 and resistant K562-TAX cell lines (IC_50_ 3.4 µM and 5.4 µM, respectively) as well as the colon cancer cell lines HCT116 and HCT116p53 (IC_50_ 3.5 µM and 3.4 µM, respectively).

**Figure 15:**
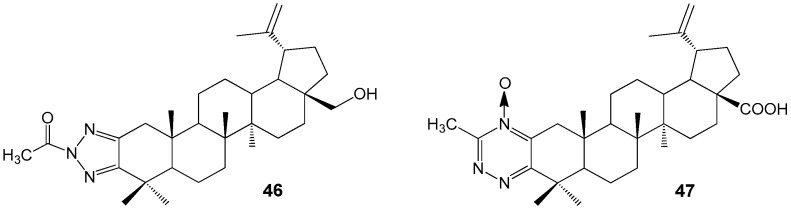
Ring-A fused azole and azine derivatives of lupanoids with anti-tumor activity.

Derivatives of lupanoids containing 1,2,3-triazol and 1,2,4-triazine groups were synthesized and their cytotoxic effects were evaluated against A549, HCT 116, HEp-2, MS, and RD TE32 cancer cell lines. N-acetyltriazole of BE **46** was found to have antiproliferative activity with IC_50_ values ranging from 2.3 to 7.5 µM against HCT 116, HEp-2, MS, and RD TE32 cell lines. The BA derivative **47** showed activity against HCT 116 and HEp-2 cell lines with IC_50_ values of 1.4 and 1.5 µM, respectively (Figure 15). Although compound **47** demonstrated higher activity, product **46** is being considered for additional investigation due to its weaker inhibitory effect on normal epithelial cell division [46].

**Figure 16:**
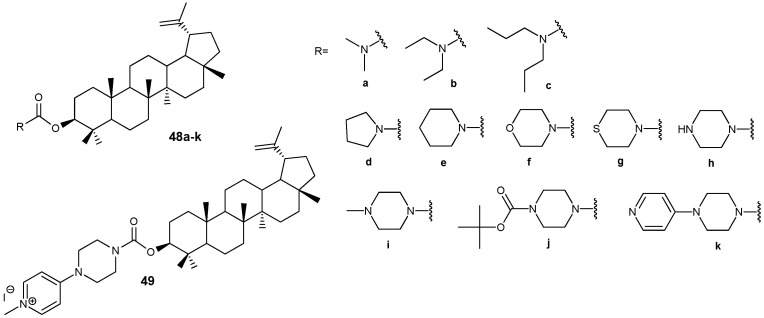
Lupeol-3-carbamates with antitumor activity.

In a series of recently synthesized lupeol-3-carbamates **48a**–**k** (Figure 16), compounds **48e**,**h**,**i**,**k** were identified, the antiproliferative activity of which (IC_50_ < 20 µM) towards three tumor cell lines A549, HepG2, MCF-7 significantly exceeded that of the parent lupeol (IC_50_ > 35 µM). The transformation of conjugate **48k** into the salt form **49** led to a significant increase in solubility (0.35 mg/mL) and activity (HepG2, IC_50_ = 3.13 µM) compared to the parent compound **48k** (HepG2, IC_50_ = 13.98 µM) [47]. According to the preliminary data obtained by the authors of this study, the antitumor effect of compound **49** is mediated by inhibition of the PI3K/AKT/mTOR signaling pathway, which initiates apoptosis in HepG2 cells.

**Figure 17:**
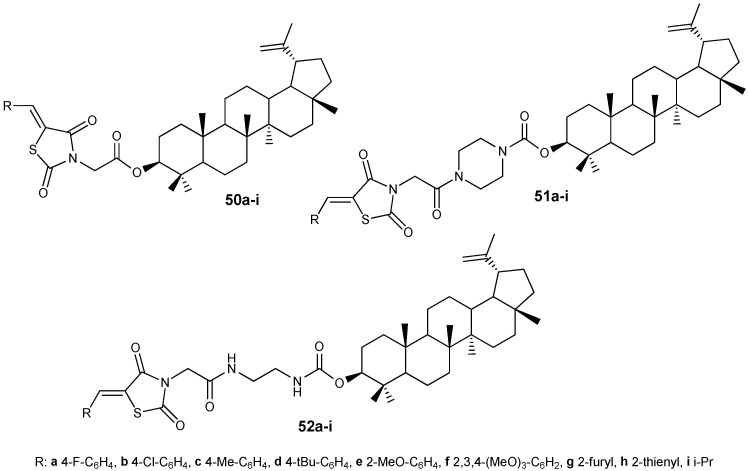
Thiazolidinedione-conjugated lupeol derivatives.

By modifying lupeol with ylidene derivatives of thiazolidinediones, long known for their antiproliferative properties [48], three groups of hybrids **50**–**52** were obtained (Figure 17), among which compound **52i** (IC_50_ 4.40 µM) was found to be the most active in inhibiting the growth of HepG2 cells, 9.9 times more potent than the effect of the original lupanoid (IC_50_ = 43.62 µM) [49].

**Figure 18:**
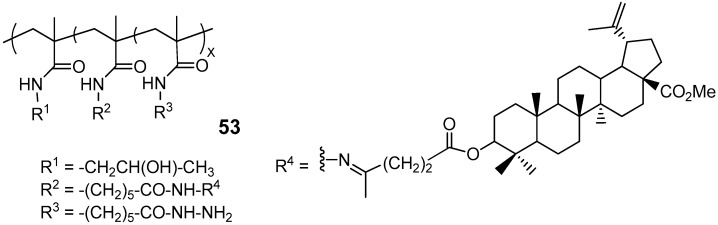
Micellar polymer-BA conjugate based on N-(2-hydroxypropyl)methacrylamide copolymer.

A few research groups have reported efforts to address the targeted delivery challenge of poorly soluble pentacyclic triterpenoid derivatives through micellar polymer conjugates of BA and soluble PEG-BA prodrugs [34,50,51]. Among these, conjugate **53** (Figure 18), featuring methylated BA levulinate at 1.0 mol%, was the sole representative from a series of BA hybrids utilizing N-(2-hydroxypropyl)methacrylamide copolymer carriers to demonstrate pH-dependent controlled release of the active substance in vitro. Esterification of the C-28 carboxylic group was essential to ensure pH-dependent controlled release of the drug candidate. Compound 53 exhibited high cytotoxicity (Table 1) in vitro against the DLD-1, HT-29 (both human colon adenocarcinoma) and HeLa cancer cell lines, outperforming both BA and its synthetic precursor, methylated BA levulinate, but slightly inferior to BA-levulinate with an unmethylated C-28 carboxyl group [50]. According to the authors of this study, the main advantage of compound **53** is that the micellar structure facilitated the accumulation of this conjugate in tumor cells, which was experimentally confirmed by fluorescence imaging in mice with two xenografted tumors.

To obtain water-soluble derivatives of BA, Dai et al. synthesized esters of this acid with multiarm-PEGs. The resulting compounds (4arm-PEG40K-BA, 8arm-PEG40K-BA, and 8arm-PEG20K-BA) contained BA at levels between 3.26–11.81 wt.% and exhibited significantly higher solubility in water compared to BA alone, increasing by 290–750 times. In vitro activity was assessed using LLC and A549 lung tumor cell lines; the 4arm-PEG40K-BA conjugate showed IC_50_ values of 37.03 and 27.19 μg/mL, respectively [51].

One of the principal challenges in effective chemotherapy for malignant tumors is the phenomenon of multidrug resistance—where cancer cells develop resistance to a range of drugs with varying chemical structures and mechanisms of action. Contributing factors include inherent genetic characteristics, enhanced DNA repair capability, defects in apoptotic pathways, alterations in target protein structures for antineoplastic agents, enzymatic drug deactivation, and the overexpression of ATP-dependent membrane transporters such as ABC proteins (ATP-binding cassette transporters). These mechanisms collectively reduce the intracellular concentration of therapeutic agents below cytotoxic levels in cancer cells [52]. The mechanism of development of multiresistance, which is mediated through ABC proteins such as P-glycoprotein (P-gp) and related proteins including MRP1, MRP2, and VSRP, has been widely studied. These proteins are identified as potential targets for pharmacological strategies addressing tumor resistance to chemotherapy. Although three generations of P-gp/MRP1 inhibitors have reached clinical trial stages, none have been approved for use as drugs in this profile [53,54]. Key challenges for their medical application include low specificity, inadequate affinity for the binding site, cross-substrate specificity between P-gp/MRP1 and CYP450 family enzymes responsible for xenobiotic metabolism, as well as interference with physiological ABC protein functions in healthy cells.

In this context, the development of conjugates combining natural compounds—which do not modulate P-gp/MRP1 activity—with synthetically derived molecules exhibiting anti-tumor properties appears to hold significant promise. Studies have demonstrated that BE, BA, and betulonic acid do not influence ABC protein activity in normal cells [21,55,56]. Conversely, the chemotherapeutic agent and immunosuppressant methotrexate (MTX) is recognized as a specific substrate for P-gp, contributing to drug resistance observed during rheumatoid arthritis treatment [57].

**Figure 19:**
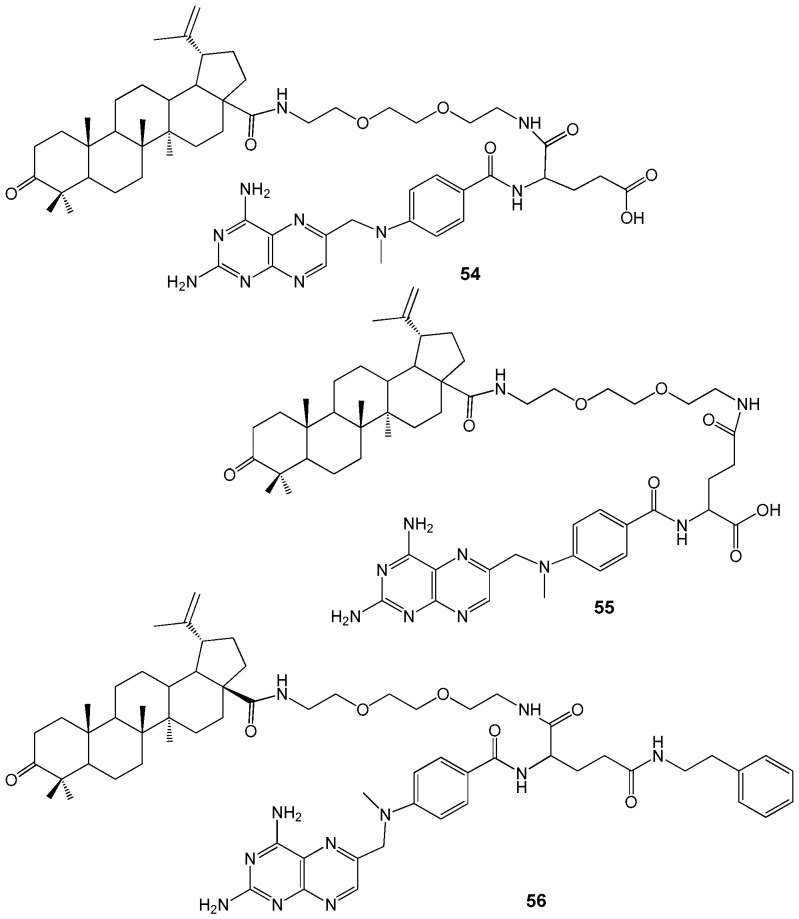
MTX-betulonic acid hybrids.

Recently, new MTX-BA hybrids **54**–**56** containing a (*tert*-butoxycarbonylamino)-3,6-dioxa-8-octanamine (Boc-DOOA) linkage were synthesized (Figure 19). Their distribution in artificial 1,2-dipalmitoylphosphatidylcholine (DPPC) membranes was evaluated by differential scanning calorimetry. The ability of compounds **54**–**56** to permeate cell membranes was tested using human colorectal adenocarcinoma (Caco-2) cells at a concentration of 10 μM [58].

MTX exhibited minimal impact on the artificial lipid membrane, whereas BA demonstrated significant membranotropic properties. Consequently, the integration of MTX-BA conjugates into the lipid membrane is primarily attributed to the lupane moiety. Conjugate **56** displayed the most substantial membranotropic effect, likely due to its highly spherical molecular geometry, although it exhibited the lowest permeability (Papp (AB) = 0.09·10^−6^ cm/s). Among the two isomeric hybrids, conjugate **54** showed a higher capacity for transmembrane diffusion in Caco cells (Papp (AB) = 0.30·10^−6^ cm/s) compared to compound **55** (Papp (AB) = 0.16·10^−6^ cm/s). Conjugate **54** has a penetrating ability similar to atenolol (Papp (AB) = 0.26·10^−6^ cm/s), but both MTX-BA hybrids **54** and **55** are less permeable than propranolol (Papp (AB) = 27.74·10^−6^ cm/s) and MTX (Papp (AB) = 0.88·10^−6^ cm/s). The reduced permeability of conjugates **54**–**56** compared to MTX may be due to differences in transport mechanisms and their higher lipophilicity, which likely leads to membrane retention.

## 3. Anti-Inflammatory Activity of Lupanoids

Chronic inflammatory processes are associated with various common diseases, including autoimmune disorders, asthma, arthritis, inflammatory bowel disease, and cardiovascular complications. These conditions can result in decreased quality of life and disability for affected individuals. Pharmacological management often involves long-term administration of nonsteroidal anti-inflammatory drugs and steroids to alleviate symptoms and support quality of life. Although these therapies are effective, they may cause adverse effects such as ulcerogenic activity, alterations in carbohydrate metabolism, hypertension, cardiac changes, and renal toxicity, particularly with prolonged use [59,60]. Consequently, identifying new substances that exhibit high anti-inflammatory activity, low toxicity, and lack the undesirable properties remains an important area of research. Betulinic acid and its derivatives are among the most frequently studied compounds with such properties in the lupanoid series, as indicated by several publications [61,62,63,64,65,66].

The anti-inflammatory properties of the natural BA conjugate with caffeic acid—pyracrenic acid (3b-(3,4-dihydroxycinnamoyl)-oxlup-20(29)-en-28oic acid), isolated from *Pyracantha crenulata*, were first documented by Otsuka H. et al. in 1981 [61]. The earliest report concerning similar activity of BA itself dates to 1995 [62]. Since that time, extensive research has been conducted on both natural and synthetic lupanoid derivatives, evaluating their anti-inflammatory effects in various in vitro and in vivo experimental models [63,64,65,66]. Studies have demonstrated that BA can reduce the activity of multiple molecules and cells implicated in the inflammatory process, including pro-inflammatory cytokines such as IL-1β, IL-6, IL-8, IL-12, and tumor necrosis factor alpha (TNFα). Additionally, BA inhibits the production of the key inflammatory mediator nitric oxide (NO) by macrophages, suppresses cyclooxygenase-2 (COX-2) expression, and consequently decreases prostaglandin E2 (PGE2) synthesis, which is associated with symptoms of inflammation such as fever, pain, and platelet aggregation [64,65].

**Figure 20:**
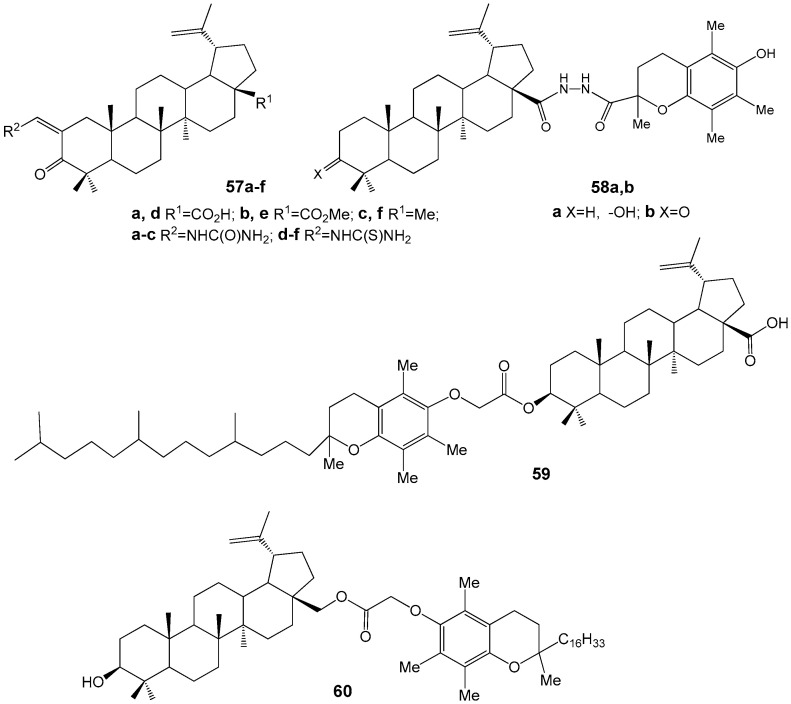
Hybrid compounds from lupane series with anti-inflammatory activity.

Betulonic acid derivatives **57a**–**f**, modified with urea and thiourea fragments (Figure 20), exhibited limited anti-inflammatory and antiulcer activities. Similarly, compounds such as betulin dinicotinate, betulin bishemi-phthalate, betulin 3,28-di-O-(2,2,3,3-tetramethylcyclopropyl)carbamate, and allobetulin 3-O-(2-(4-chlorophenyl)-3-methyl)-butanoate, synthesized by the same research group, demonstrated moderate anti-inflammatory effects [66].

Hybrids of BE, BA, and betulonic acid (compounds **58**–**60**) containing chroman fragments (see Figure 20) exhibit both antioxidant properties, attributable to the chroman moiety, and the ability to selectively inhibit nitric oxide (NO) production in activated macrophages without affecting arginase activity [67].

**Figure 21:**
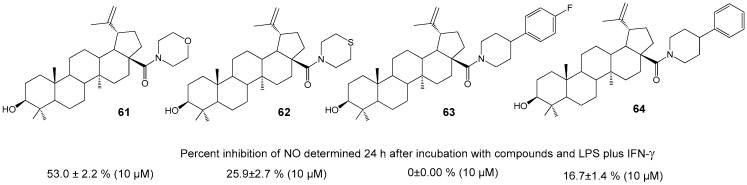
BA derivatives with immunomodulatory and anti-inflammatory properties.

Meira C.S. et al. synthesized cyclic amides of BA **61**–**64** (Figure 21) and studied their immune-modulatory [68] and anti-inflammatory properties [65]. Morpholine derivative **61** was tested in vitro for its inhibitory effect on NO production in activated macrophages, with results comparable to reference drug dexamethasone (57.6 ± 3.2% at 10 µM), as well as for its impact on reducing expression of TNF and NF-κB inflammation mediators [65]. The compound’s anti-inflammatory activity was also examined in an experimental model of endotoxic shock, where it reduced edema formation induced by bovine serum albumin. BA hybrid **61**, administered at doses of 1 and 10 mg/kg *per os*, lowered heart inflammation and fibrosis in a C57BL/6 model of chronic cardiomyopathy related to Chagas disease. The study associated these effects with the induction of IL-10 and M2 macrophage polarization [68].

**Figure 22:**
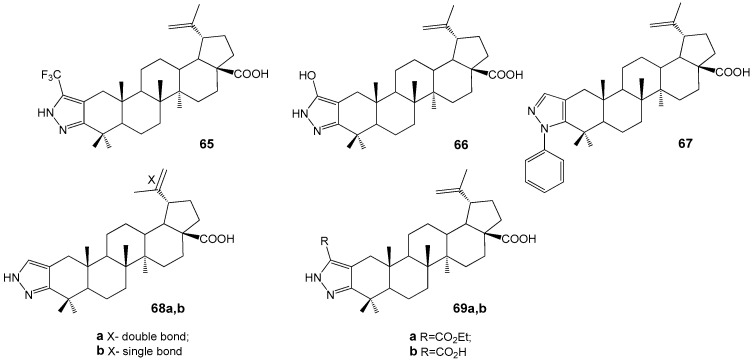
BA-pyrazole hybrides with anti-inflammatory properties.

Pyrazole derivatives **68a**,**b** (Figure 22) showed strong anti-inflammatory and potent inhibitory activity of osteoclastogenesis and bone resorption (100% at 10 µM) [69]. They were synthesized from betulonic acid via diketone formation and condensation with hydrazines, alongside hybrids **65**–**69**. Pyrazole-BA conjugate **68a** demonstrated significant anti-inflammatory effects in a collagen-induced arthritis model [70].

**Figure 23:**
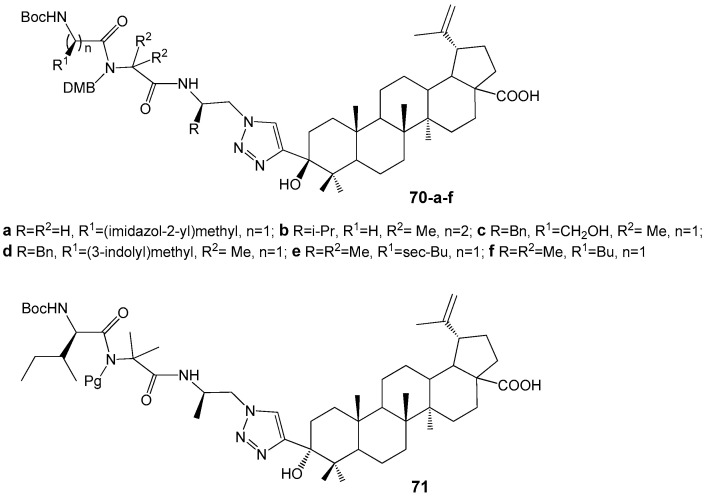
BA-peptide hybrids with high anti-inflammatory activity.

Govdi A. et al. reported the synthesis of new BA-peptide hybrids using a 1,2,3-triazole linker [71]. The target compounds **70a**–**f** and **71** (Figure 23) were produced via Cu^+^-catalyzed click-reactions with high yields. Compounds **70a**,**b**,**d**,**e** demonstrated anti-inflammatory activity comparable to the reference drug indomethacin.

## 4. Antiviral Activity of Lupanoids

The development of effective antiviral agents represents a significant challenge in both medicinal chemistry and pharmacology. This complexity arises from viruses’ reliance on the host cell’s reproductive machinery for replication and their high mutation rates. According to the World Health Organization, some of the most prevalent and hazardous viral infections globally include rabies virus, HIV, hepatitis C virus (HCV), influenza A viruses (H5N1 and H7N9), coronaviruses (SARS-CoV, MERS-CoV, COVID-19), as well as tropical region viruses such as Ebola (EBOV), Marburg, Lassa, and Zika (ZIKV) [72,73,74,75,76]. The emergence of the COVID-19 pandemic, the spread of ZIKV throughout Central and South America, and outbreaks of EBOV in Africa have further driven the accelerated advancement of antiviral drug development. Plant metabolites are considered promising sources for the development of basic structures used in designing such agents. Between 1981 and 2014, more than 40% of antiviral drugs in clinical use were either natural products or derived from natural product prototypes [77]. Researchers have investigated lupanoids as molecular platforms for antiviral agent design following Fujioka et al.’s discovery that BA from the leaves and bark of *Syzigium claviflorum* displayed notable activity against HIV (EC_50_ = 1.4 µM) [78]. Additionally, pentacyclic triterpenoids have high lipophilicity, which enables these molecules to interact with viral capsids and potentially inhibit the entry of viral particles into host cells [79].

### 4.1. BA and BE Hybrids with Anti-HIV Activity

**Figure 24:**
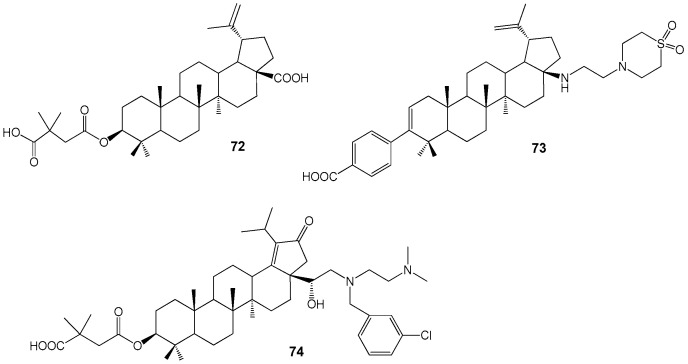
Novel HIV-1 maturation inhibitors.

The BA derivative Bevirimat (BVM) **72** is an anti-HIV compound classified as a maturation inhibitor (Figure 24) [80,81]. It demonstrates anti-HIV activity (IC90 22.2 ng/mL in vitro) against both primary and drug-resistant HIV isolates [82], functioning by binding to the CA/SP1 cleavage site of the viral Gag polyprotein and preventing formation of the CA/p24 capsid protein from its precursor CA-SP1/p25. Due to baseline Gag polymorphisms reducing HIV-1 sensitivity to BVM, its development was discontinued in 2010 [83]. Subsequently, additional lupanoid derivatives, BMS-955176 **73** and GSK-2838232 **74**, were investigated as oral treatments for HIV infection [84,85,86,87].

**Figure 25:**
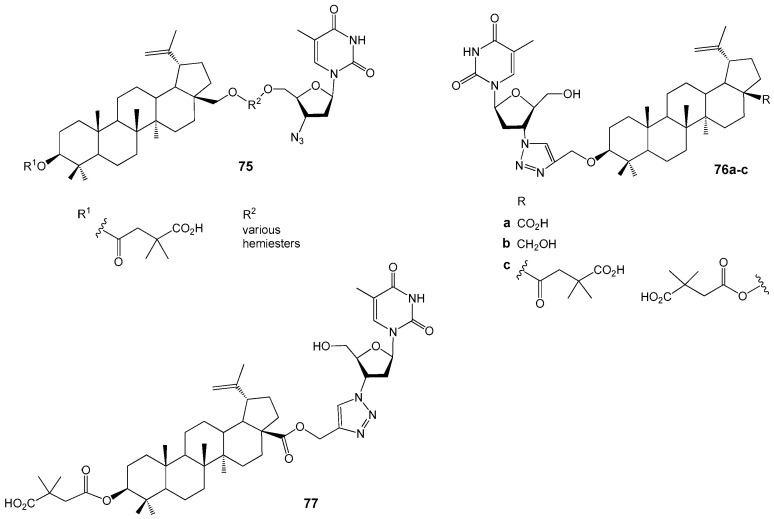
BE-AZT and BA-AZT conjugates with anti-HIV activity.

Xiong J. et al. linked BVM **72** to AZT using a short ester linker, resulting in the synthesis of 14 conjugates, nine of which demonstrated potent anti-HIV activity with EC_50_ values ranging from 40 to 98 nM in HIV-1 (NL4-3)-infected MT-4 cells (Figure 25). The most effective compound, **75** (EC_50_ = 0.040 µM), exhibited markedly greater inhibition of viral replication compared to the parent BVM (EC_50_ = 0.096 µM). In contrast, conjugates modified at the 3-OH group **76a**–**c** displayed reduced activity relative to compound **75** [88]. Additionally, BVM conjugated with AZT at the C-28 position of the lupane scaffold via click chemistry **77** and its propargylic ester precursor also showed substantial anti-HIV activity (EC_50_ values of 0.10 µM and 67 nM, respectively), comparable to that of AZT (EC_50_ = 0.10 µM) [89].

**Figure 26:**
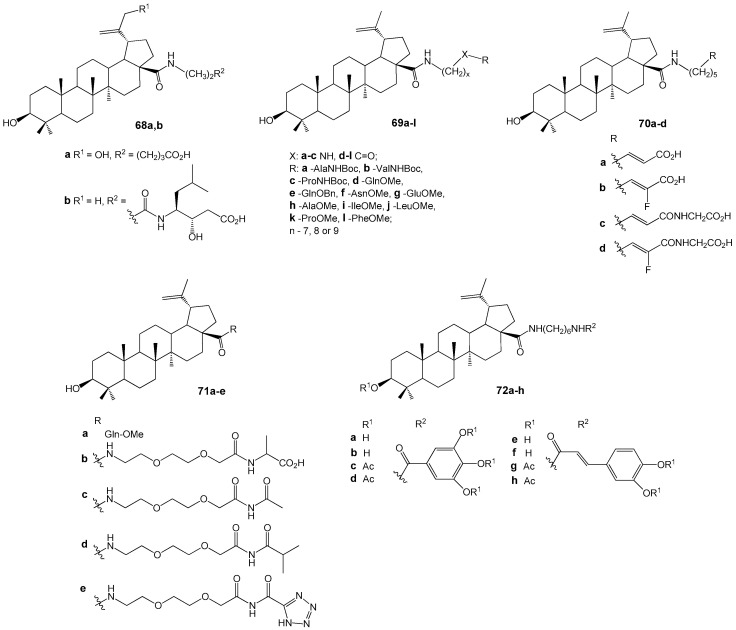
BA amides—inhibitors of HIV virus entry.

Studies [88,89] show that attaching AZT to the C-28 position of the lupane scaffold enhances anti-HIV activity, while a free azide group or its presence in a 1,2,3-triazole linker does not significantly impact activity. Forming amino acid conjugates with the BA carboxylic group (Figure 26) yields strong HIV entry inhibitors, such as hybrids **78a**,**b** (EC_50_ ~ 50 nM) [90] and **79**–**81** [91]. Glutamine ester derivatives **78d**,**e**,**g** display high activity against BVM-resistant viruses. Although many compounds **79a**–**f**,**j** had EC_50_ values between 0.04 µM and 0.1 µM, **79d**,**e**,**j** were prioritized for their favorable properties, including microsomal stability [91].

Conjugates of BA with gallic or caffeic acid **82a**–**h** (Figure 26) were studied as inhibitors of HIV-1 gp41 fusion core formation [92]. Several galloyl derivatives demonstrated activity approximately five times greater than that of BA, suggesting their potential as lead structures for the development of HIV-fusion/entry inhibitors.

**Figure 27:**
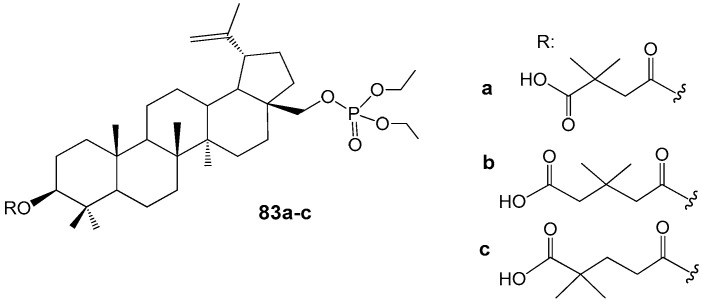
BE phosphate derivatives with anti-HIV activity.

Recent studies have demonstrated that readily accessible phosphate derivatives of 3-carboxyacylbetulin (**83a**–**c**, Figure 27) exhibit in vitro anti-HIV activity with IC_50_ values ranging from approximately 0.02 to 0.22 µM [93]. The most potent compound identified is the phosphate analog of BVM (**83a**, IC_50_ = 0.02 µM), which inhibits viral replication at levels comparable to BVM, while displaying higher selectivity (SI = 1250 for **83a** compared to SI = 967 for BVM). Molecular docking analyses conducted by the authors [90] indicate that this hybrid molecule possesses additional binding sites with the CA-CTD-SP1 fragment of the Gag protein, attributable to the presence of phosphate groups within its structure. Consequently, compound **83a** is regarded as a promising lead among HIV-1 maturation inhibitors, with potential efficacy surpassing that of BVM.

### 4.2. BA and BE Hybrids with Anti-Influenza Virus Activity

**Figure 28:**
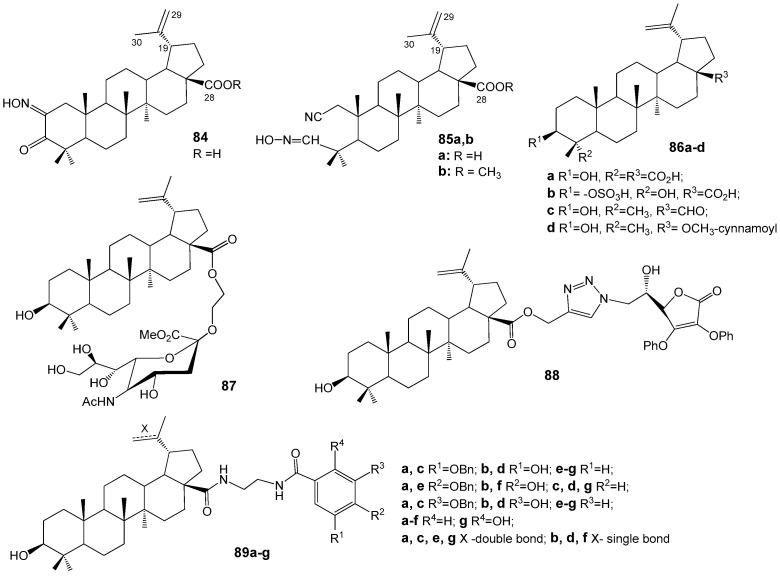
Lupane derivatives with activity against influenza A virus and HIV-1.

Grishko V.V. et al. [94] synthesized modified triterpenoids derived from lupane and oleanane series and evaluated their antiviral properties in vitro against influenza virus A/FPV/Rostock/34 (H7N1) and HIV-1. Lupane triterpenoids ketoxime **84** and 2,3-secoaldoximes **85a**,**b** (Figure 28) exhibited EC_50_ values of 12.9–18.2 µM against influenza A virus. Compounds containing an oxime fragment and a free C-28 carboxylic group, such as triterpene oximes **84** and **85a**, demonstrated suppression of HIV-1 replication in vitro with an EC_50_ of 0.06 µM.

BA, isolated from the Jujube tree (*Zizyphus jujube* Mill), exhibited antiviral properties against the influenza A/PR/8 virus both in vitro and in vivo [95]. Derivatives of BA, specifically compounds **86a**,**b** (Figure 28) sourced from Chinese herbal medicine *Schefflera heptaphylla*, demonstrated inhibitory effects on H1N1 influenza A virus, with IC_50_ values of 32.2 and 58.4 µM, respectively [96]. BEA (**86c**) derived from *Alnus japonica* showed anti-influenza activity against the influenza A/Korea/KBNP-0028/2000 strain (IC_50_ = 12.5 µg/mL); however, its pronounced cytotoxicity (CC_50_ = 24.3 µg/mL) limits its potential for further development [97]. According to study [98], 28-*ortho*-OCH_3_-cynnamoylbetulin (**86d**) demonstrated potent efficacy (EC_50_ = 2 µg/mL) against the H1N1 influenza A/California/07/2009 virus, coupled with low cytotoxicity and a selectivity index greater than 100.

Zhou D. et al. [99] synthesized BA hybrids with sialic acid derivatives and evaluated their potential as anti-influenza agents. Notably, compound **87** (Figure 28) demonstrated the highest activity against the A/WSN/33 (H1N1) virus, exhibiting an IC_50_ value of 41.2 µM, which was comparable to that of the reference drug oseltamivir. Data from hemagglutination inhibition assays, binding stability measurements of compound **87** with hemagglutinin protein (HA) using surface plasmon resonance (Kd = 17 µM), and molecular docking studies on the HA protein model collectively support the hypothesis that the synthesized conjugate may function as an inhibitor of influenza virus entry by preventing the interaction between viral HA and specific receptors on host cells.

The BA conjugate with *L*-ascorbic acid **88** from the series of analogous hybrids of various triterpenoids demonstrated the most significant anti-influenza activity (IC_50_ = 8.7 µM) and exhibited favorable selectivity [100]. Within the collection of BA conjugates containing different hydroxybenzoic acid derivatives **89a**–**g**, compound **89g** displayed the highest potency (IC_50_ = 5.8 µM) against influenza A/WSN/33 (H1N1), coupled with low cytotoxicity. The authors of the study [101] propose that the anti-influenza mechanism of this compound involves binding to capsid HA, thereby preventing viral entry into host cells.

### 4.3. Anti-SARS Activity of Lupanoids

The global outbreak of severe acute respiratory syndrome (SARS), an infectious respiratory illness caused by the coronavirus (CoV), garnered significant international attention following its emergence in China during 2002–2003 [79]. Wen et al. conducted an assessment of 221 phytocompounds for their efficacy against SARS-CoV [102]. The study found that the EC_50_ value for BA exceeded 10 µM against SARS-CoV, while betulonic acid demonstrated an EC_50_ value of 0.63 µM, which is 2.6 times lower than the positive control, valinomycin (EC_50_ = 1.63 µM). Both compounds exhibited CC_50_ values greater than 100 µM. The selectivity index (SI) of betulonic acid was calculated at 180, signifying potent inhibition of viral replication with minimal cytotoxicity. These acids were evaluated using an inhibition assay targeting 3CL protease, where BA displayed inhibitory activity with an IC_50_ value of 10 µM and a Ki of 8.2 ± 0.7 µM, consistent with a competitive inhibition mechanism. At the same time, the IC_50_ of betulonic acid exceeded 100 µM. Molecular docking analysis indicated that BA occupies the binding pocket of SARS-CoV 3CL protease, with enhanced binding attributed to a hydrogen bond between the C3-OH group of BA and the C=O of Thr24 within the enzyme. However, aside from hydrophobic interactions, betulonic acid did not establish additional intermolecular bonds within the binding site. The researchers propose that the observed anti-SARS activity may result from a combination of two distinct mechanisms: inhibition of 3CL protease and blockade of SARS-CoV entry during the post-binding step with the host cell membrane, which is recognized as an important and general antiviral mechanism for pentacyclic terpenoids.

### 4.4. Activity of Lupanoids Against HBV and HSV

The traditional Chinese herb *Pulsatilla chinensis* has been observed to have activity against the *Hepatitis B virus* (HBV). BA and its derivatives were identified as anti-HBV agents among 30 metabolites isolated from this plant [79,103]. Research by Yao D et al. [104] indicates that BA can inhibit superoxide dismutase-2 in liver tissue and thereby increase the production of reactive oxygen species, which may contribute to inhibiting HBV replication in vitro and in vivo.

HSV-1 is classified within the *Herpesviridae* family, a group associated with significant public health concerns. According to the World Health Organization, 67% of the global population under 50 years of age are infected with this virus [105]. Current research into anti-HSV agents includes the evaluation of triterpenoids from the lupane series and their derivatives [88]. Gong Y et al. [106] demonstrated that BE and acyclovir exhibit synergistic anti-HSV-1 effects at concentrations above 0.9 µM and 4.4 µM, respectively. Comparable synergy was observed against HSV-2 with acyclovir and BE administered at 2.0 µM and 19.0 µM, respectively. BE displays activity against both HSV-1 (EC_50_ = 0.40 µg/mL) and HSV-2 (EC_50_ = 4.15 µg/mL). In comparison, acyclovir’s EC_50_ values are 0.28 μg/mL for HSV-1 and 0.88 μg/mL for HSV-2. The observed synergistic antiviral effect suggests a distinct mechanism of action for BE compared to acyclovir. Navid et al. [107] further studied this mechanism, revealing that BE inhibits both acyclovir-sensitive and acyclovir-resistant clinical strains of HSV-1 during the early stage of infection.

**Figure 29:**
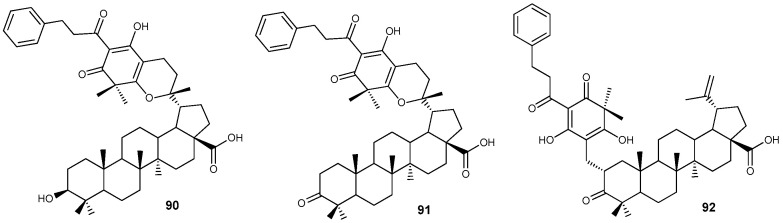
Phloroglucinol conjugates of BA and betulonic acid.

High anti-HSV-1 activity was recently revealed for compounds **91** and **92** (IC_50_ ~ 6 µM/L) from a group of natural phloroglucinol conjugates of BA and betulonic acid **90**–**92** (Figure 29) isolated from *Leptospermum scoparium* of the *Myrtaceae* family [108].

ZIKV is an arbovirus belonging to the *Flaviviridae* family. Since its identification in the late 1940s, ZIKV was originally not regarded as a significant threat to humans, in contrast to other family members such as West Nile fever, yellow fever virus, dengue virus, and Japanese encephalitis virus, which are known to cause severe diseases in tropical regions [109]. However, perceptions of ZIKV shifted substantially following a rise in cases of Guillain-Barré syndrome and microcephaly in newborns reported in several countries across Africa, Asia, and, notably, South America in 2015 [110]. Subsequent investigations established Zika virus as the etiological agent responsible for these conditions, with evidence indicating its capacity to infect neural progenitor cells (NPCs), disrupt the expression of cell cycle-related proteins, induce apoptosis, and impair neurogenesis [111]. Currently, there is no approved vaccine or specific therapy for the prevention or treatment of ZIKV infection. As a result, researchers are investigating substances with potential anti-ZIKV activity. Navabi, S. et al. [112] noted that BA may possess neuroprotective properties. Studies have shown that BA can cross the blood–brain barrier, suggesting its potential as a molecule for addressing CNS disorders.

Cavalcante B. et al. [113] reported that the neuroprotective anti-ZIKV effect of BA is dose-dependent, as demonstrated in experiments using 3D cultures with brain organoids derived from neuronal progenitor cells (NPCs). At a concentration of 50 µM, BA was not toxic to uninfected NPCs but resulted in notable cell loss in ZIKV-infected cultures. The findings suggest that BA is a suitable base structure for further research into anti-ZIKV agents, and ongoing studies may identify new BA derivatives that protect ZIKV-infected NPCs from cell death.

## 5. Antibacterial, Antifungal and Antiparasitic Properties of Lupanoids

Antibacterial resistance and the proliferation of multidrug-resistant bacterial strains present significant challenges on a global scale. Principal contributing factors include microbial evolution through natural selection owing to prolonged use of antibacterial agents, as well as the inappropriate application of anti-infective substances within medicine, agriculture, and the food industry. The World Health Organization (WHO) projects that, by 2050, infections caused by antibiotic-resistant bacteria could result in approximately 10 million deaths annually [114]. The ESKAPE group of microorganisms (*Enterococcus faecium*, *Staphylococcus aureus*, *Klebsiella pneumoniae*, *Acinetobacter baumanii*, *Pseudomonas aeruginosa*, *Enterobacter* spp.), characterized by their elevated drug resistance, are identified by WHO as primary contributors to nosocomial infections and increased mortality rates [115]. Of particular concern is the spread of carbapenem-resistant Gram-negative bacteria from this group (*Klebsiella pneumoniae*, *Pseudomonas aeruginosa*, *Acinetobacter baumanii*, *Enterobacter* spp.), since carbapenems are often considered antibiotics of “last resort” for hospitalized patients. Additionally, some carbapenem-resistant organisms have demonstrated resistance to colistin, which is recommended for the treatment of superinfections [116,117]. These developments underscore the necessity for effective solutions to antimicrobial resistance. One promising approach involves identifying biologically active compounds with novel mechanisms of action against bacterial cells, thereby prompting further investigation into new molecular targets for these agents.

### 5.1. Activity of Lupanoids Against ESKAPE Group Bacteria

Chung P.Y. et al. reported [118] on the antimicrobial properties of BA and betulinaldehyde (BEA), which were isolated from the bark of *Callicarpa farinosa*. The study found that both compounds demonstrated low activity against standard and clinical strains of methicillin-resistant (MRSA) and methicillin-sensitive *Staphylococcus aureus* (MSSA). BA showed higher activity (MIC = 64 μg/mL) compared to BEA (MIC 512 μg/mL for MRSA and 256 μg/mL for MSSA). The authors attributed these findings to the high lipophilicity of BA and BEA (log *p* values of 8.94 and 9.07, respectively), which may reduce the rate at which the compounds penetrate bacterial cells.

**Figure 30:**
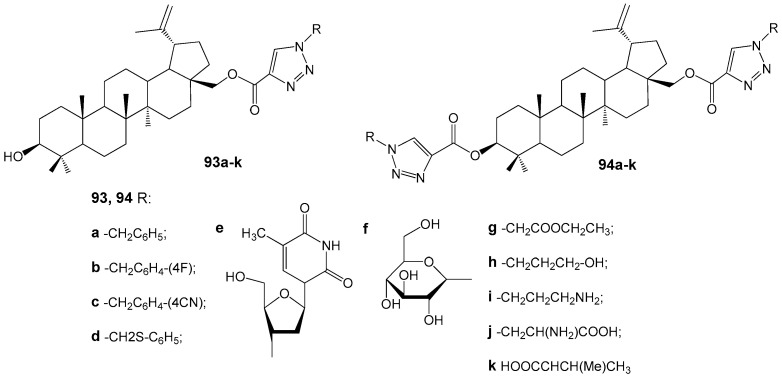
BE derivatives with potential antibacterial activity.

Transcriptome analyses conducted with reference strains MRSA ATCC 43300 and MSSA ATCC 29213 identified potential targets of BA and/or BEA within the cell division process, including fatty acid biosynthesis, ABC transport systems, peptidoglycan biosynthesis, aminoacyl-tRNA synthetase, and ribosomes [118]. Notably, BA and BEA demonstrated activity against targets that are not present in human cells, indicating their promise as scaffolds for the development of novel therapeutic agents to treat *S. aureus* infections. Furthermore, the broad range of targets increases the practical utility of BA and BEA derivatives, as simultaneous effects on multiple pathways can reduce the likelihood of bacterial resistance. The antibacterial properties of 1,2,3-triazole BE conjugates **93a**–**k** and **94a**–**k** (Figure 30) were evaluated against both Gram-positive and Gram-negative bacterial strains [119]. Among these compounds, only **93e** exhibited antibacterial activity against *Klebsiella pneumoniae* and *Escherichia coli*, with MIC values of 0.95 and 1.95 µM, respectively.

**Figure 31:**
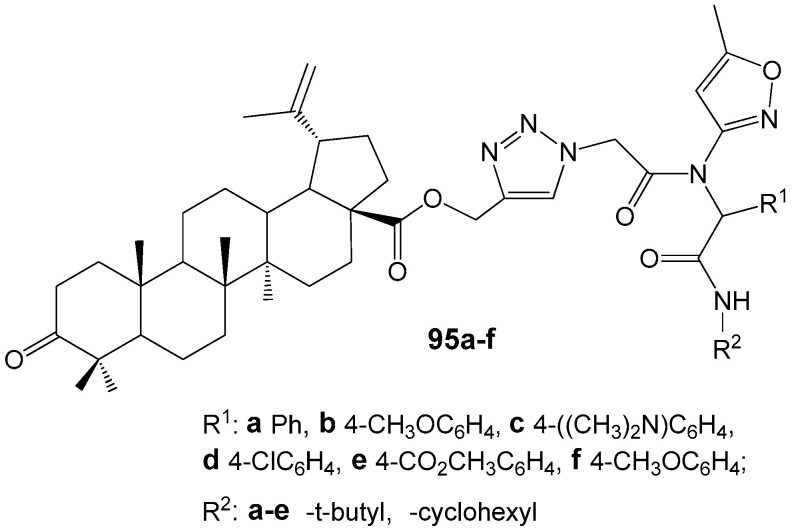
Betulonic acid derivatives with peptidomimetic moiety as potential antibacterial agents.

Recently were reported the synthesis and study of the microbiological properties of new betulonic acid derivatives **95** with peptidomimetic moiety containing 1,2,3-triazole ring at the C-28 position (Figure 31). The synthesis of hybrid molecules was carried out by a three-step transformation. The first stage was Ugi four-component reaction, the second—obtaining betulonic acid propargyl esters and the last step was Cu-catalyzed cycloaddition of peptidestructured azides to betulonic acid propargyl esters, resulting in the construction of target molecules [120]. Experiments with Gram-negative cultures *Escherichia coli* (ATCC25922), *Pseudomonas aeruginosa* (ATCC27853) and Gram-positive strains *Bacillus subtilis* (ATCC6633), *Staphylococcus aureus* (ATCC25923) showed that hybrids **95a**–**f** did not exhibit any significant toxic effects towards bacteria. This result was attributed to the extremely low water solubility of the studied compounds.

### 5.2. Lupanoids with Anti-Tuberculosis and Anti-Fungal Activity

Tuberculosis is the cause of death for 2 million people on our planet every year. The spreading of this disease is aggravated by the presence of multi-resistant forms of *Mycobacterium tuberculosis* (MBT) [121,122]. In this regard, the following information about the anti-tuberculosis properties of lupanoids deserves attention: BE inhibited the growth of MTB with MIC values of 25–30 µg/mL [123,124], BEA showed activity with MIC 25 mg/mL [124], BA exhibited low antimycobacterial activity MIC in the range of 25 µg/mL to >100 µg/mL according to different sources [124,125,126,127]. The change of the orientation of the hydroxyl group at C-3 from β to α location significantly increased the activity of lupeol (MIC > 64 µg/mL) [127]. It is believed that the activity of pentacyclic triterpenoids against MBT is associated with their high lipophilicity, due to which these compounds penetrate the lipid rich bacterial cell wall.

**Figure 32:**
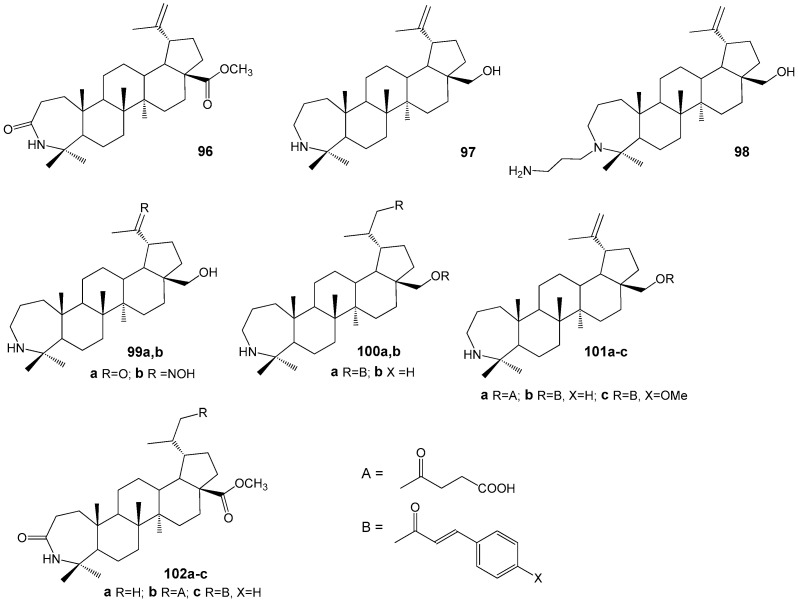
Azepanolupanoids as inhibitors of MTB.

The author of the research [128] synthesized a series of azepanotriterpenoids (Figure 32) as a novel class of inhibitors of MTB. From the obtained compounds **96**–**102** A-azepano-28-cinnamoyloxybetulin **101b** in vitro assay showed the highest MIC 2 and MBC 4 µM against MTB H37Rv (reference drugs: rifampicin MIC 0.04 µM; isoniazid MIC 0.31 µM) and MICs 4, 1 and 1 µM against isoniazid, rifampicin and ofloxacin resistant strains, respectively.

### 5.3. Antiplasmodial, Antileishmanial, Antitrypanosomal and Antischistosomiasis Properties of Lupanoids

Innocente A.M. et al. reported the synthesis and biological assessment of BA C-3 acetate, which was modified at position C-28 with an aminopropyl piperazine moiety [129]. The compound N-{3-[4-(3-aminopropyl)piperazinyl]propyl}-3-acetylbetulinamide exhibited minimum inhibitory concentrations (MIC) against pathogenic yeasts ranging from 4 to 16 µg/mL and demonstrated fungicidal activity at concentrations between 8 and 128 µg/mL. The antifungal properties of this molecule were comparable to those of terbinafine, a well-established drug for treating onychomycosis caused by dermatophytes or Candida species, supporting its consideration as a lead compound for the development of new antifungal agents.

Malaria, leishmaniasis, and trypanosomiasis are prevalent in tropical regions and present significant challenges to public health [130,131,132,133,134]. The repertoire of available antiparasitic medications remains limited. Currently, chloroquine (CQ) and artemisinin derivatives represent the most effective treatments for malaria, while amphotericin B, paromomycin, miltefosine, pentamidine, and sodium stibogluconate are primary agents against leishmaniasis. For human trypanosomiasis, therapeutic options include pentamidine, benznidazole, and nifurtimox [135].

BA, BE, and BEA have been shown to inhibit the growth of the malaria pathogen *P. falciparum*, exhibiting IC_50_ values between 7 and 28 μM. The antiplasmodial activity of lupanoids is believed to result from their incorporation into the erythrocyte lipid bilayer, leading to alterations of cholesterol-rich membrane rafts through the formation of hydrogen bonds, thereby disrupting parasite vacuolization [136]. These findings provide a foundation for modifying BA and BE with the aim of developing highly potent antimalarial agents.

**Figure 33:**
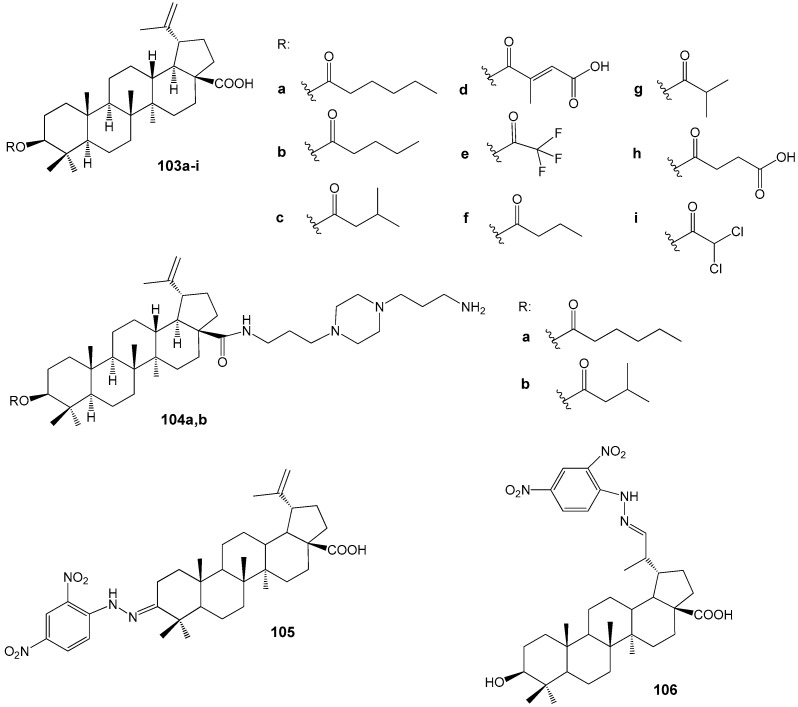
BA hybrids with antimalarial properties.

Da Silva G.N. et al. carried out experimental studies demonstrating that among the C-3 esterified BA derivatives **103f** (IC_50_ = 5 μM) and **103g** (IC_50_ = 8 μM) (Figure 33) exhibited the highest efficacy against CQ-sensitive *P. falciparum* 3D7. These compounds displayed no cytotoxicity towards the HEK293T cell line and were two to four times more active than the parent BA (IC_50_ = 18 μM) [137].

Subsequent analysis of the antiplasmodial activity of esters **103f**,**g**, along with derivatives featuring methoxy and imidazole substituents in the carboxyl group against the CQ-resistant *P. falciparum* W2 strain, led the researchers [138] to determine that structural modification at the C-3 position more effectively enhances the antimalarial properties of the BA skeleton than simultaneous modifications at both C-3 and C-28 positions. In these assays, the 3β-butanoyl BA derivative **103f** demonstrated an IC_50_ value of 3.4 μM. Furthermore, docking studies indicated a potential interaction between ester **103f** and the *Plasmodium* protease PfSUB1. The piperazinyl substituted BA **104a** (IC_50_ = 1 µM) and **104b** (IC_50_ = 4 µM) (Figure 33) exhibited antiprotazoal activity against the *P. falciparum* chloroquine sensitive 3D7 strain [139,140]. The 2,4-dinitrophenylhydrazone derivatives of BA, specifically compound **105** (IC_50_ = 15.3 µM) and **106** (IC_50_ = 10.2 µM) as illustrated in Figure 33, demonstrated moderate activity that exceeded that of the parent BA (IC_50_ = 38.8 µM) when tested against the chloroquine-resistant *P. falciparum* W2 strain [141].

**Figure 34:**
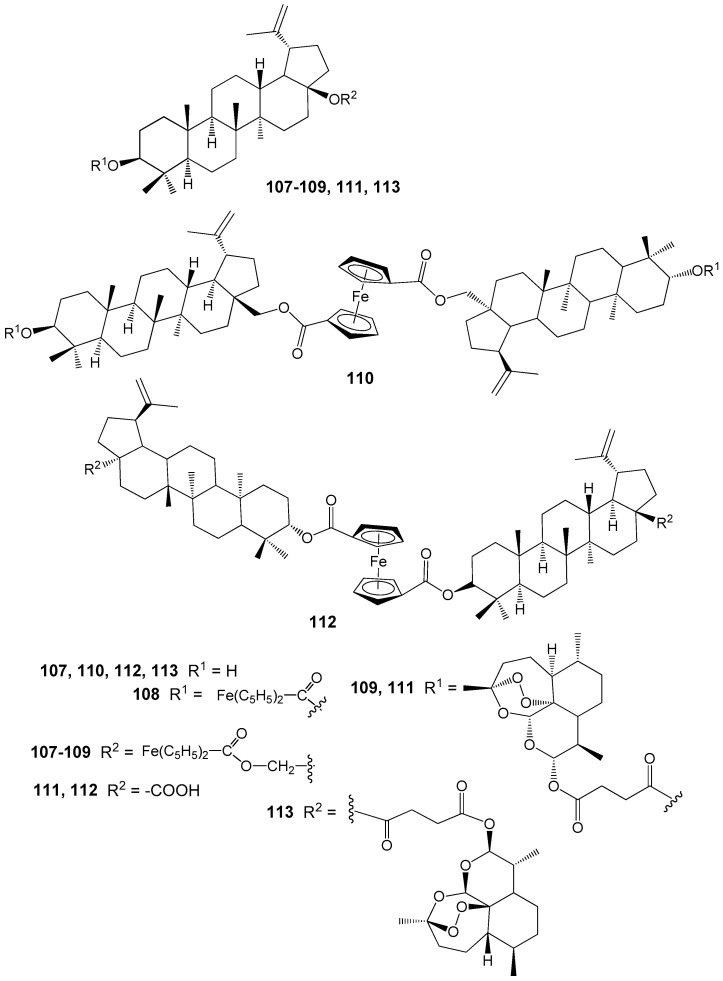
BE and BA hybrids with ferrocene and artesunic acid.

Among the BE and BA hybrids **107**–**113** containing ferrocene and/or artesunic acid moieties (Figure 34), compounds **110** (IC_50_ = 0.26 μM) and **113** (IC_50_ = 0.09 μM), all based on artesunic acid, demonstrated the highest antimalarial activity. The IC_50_ values for the parent compounds BE, BA, and artesunic acid were 3.94 μM, 1.42 μM, and 0.0097 μM, respectively. Thus, modification of the C-3 hydroxyl group in the lupane platform with an artesunic acid residue significantly enhances the antiparasitic activity. However, compound **113** is still inferior in activity to artesunic acid [142].

BEA was the first compound from the lupane family to demonstrate in vitro activity against *Leishmania amazonensis* amastigote infection, achieving 88% inhibition at a concentration of 136 µM; however, it was found to be highly toxic [135]. BA (IC_50_ = 87.5 µM), BA acetate (IC_50_ = 44.9 µM), and betulonic *a*cid (IC_50_ = 51.2 µM) also exhibited weak activity against *L. amazonensis* [143].

**Figure 35:**
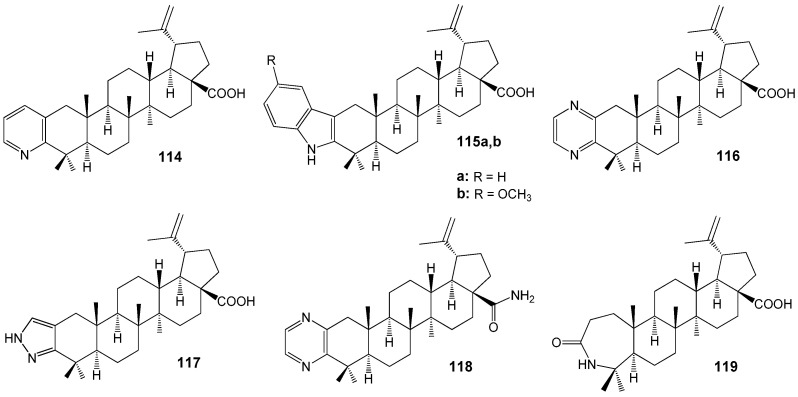
BA hybrids with antileishmanial activity against *L. donovani*.

Among the heterocyclic derivatives of BA **114**–**119** (Figure 35), compounds **118** (IC_50_ = 13.2 µM) and **119** (IC_50_ = 4.3 µM) exhibited the highest antileishmanial activity against axenic amastigotes of *L. donovani*. Azepinone **119** demonstrated the greatest selectivity (SI = 12.9) among all agents reported in this study [144].

**Figure 36:**
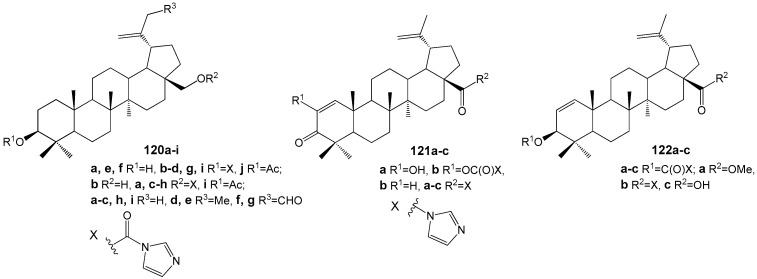
BE and BA conjugates with activity against *L. infantum*.

Sousa et al. synthesized a series of conjugates of BA and BE with imidazole, resulting in compounds **120**–**122** (Figure 36), which were subsequently evaluated against *L. infantum* [145]. Among these, **120f** and **121b** exhibited the most potent activity (IC_50_ values of 51 and 26 µM, respectively) alongside minimal toxicity, indicating their potential as candidates for further antileishmanial drug development. Notably, these compounds also demonstrated a synergistic effect when combined with miltefosine—a current antileishmanial therapeutic—reducing the IC_50_ of **121b** to 6 µM. Drawing on previous findings regarding the anti-tumor properties of imidazole conjugates of BE and BA, attributed to DNA topoisomerase inhibition in human cancer cell lines [43], the authors suggest that *Leishmania* DNA topoisomerases may serve as possible molecular targets for compounds **120f** and **121b**.

Acylation of the 3-OH group in BA, followed by allylic bromination using NBS, produced 3β-acetoxy-30-bromo-lup-20(29)-en-28-oic acid. At a concentration of 100 μM, this compound inhibited the viability of *L. amazonensis* promastigote forms by 47.41%. Introduction of a bromine atom into the triterpene scaffold enhanced antileishmanial activity, as the parent compound BA was ineffective at 100 μM under the tested conditions [146].

Although the number of lupane derivatives identified as active against leishmaniasis pathogens remains limited, and it is too early to assess their efficacy prior to clinical trials, further investigation in this area is warranted. Notably, heterocyclic derivatives—particularly when used in conjunction with established therapies—exhibit promising results.

**Figure 37:**
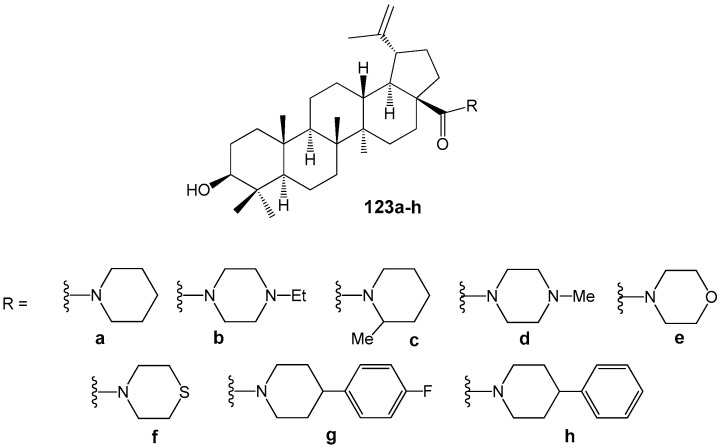
BA heterocyclic amides **123a**–**h** with antitrypanosomal properties.

BA is also present among metabolites of the African plant *Strychnos spinose* Lam., which has been traditionally used to treat trypanosomiasis. BA demonstrated low activity (IC_50_ = 32.6 µM) against bloodstream forms of *Trypanosoma brucei* and (IC_50_ = 50.0 µM) against epimastigote forms of *T. cruzi* Tulahuen strain [147]. According to these results and considering that modification of the carboxyl group in BA can increase antiparasitic activity [146], researchers synthesized a series of cyclic amides of BA (Figure 37) and evaluated their anti*-Trypanosoma cruz*i activity and selectivity [148]. Compounds **123e** (IC_50_ = 1.8 µM; SI = 17.3), **123f** (IC_50_ = 5.4 µM; SI = 5.3), and **123h** (IC_50_ = 5.0 µM; SI = 10.7) showed greater effects compared to BA (IC_50_ = 19.5 µM; SI = 1). Additionally, compound **123e** was identified as a selective agent inducing necrosis in *T. cruzi* cells. When morpholine hybrid **123e** was combined with benznidazole, a drug used for Chagas disease treatment, a synergistic effect was observed.

**Figure 38:**
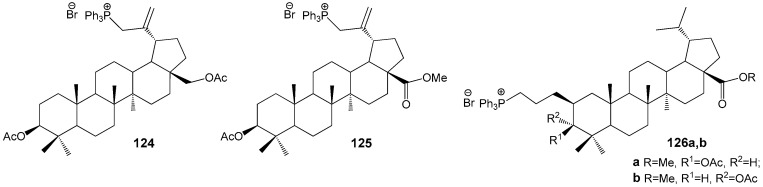
BE and BA derivatives effective against schistosomiasis.

Schistosomiasis, a significant health concern in tropical regions, is caused by the blood flukes of the species *Schistosoma mansoni*. According to WHO’s 2021 report, an estimated 251.4 million individuals across Africa, South and Central America, and the Middle East are affected by this disease [149]. Transmission occurs through contact with water contaminated by these parasites. The pressing need for new pharmaceutical agents against schistosomiasis arises from the limited availability of effective drugs and the rapid emergence of parasite resistance. Spivak et al. conducted both in vitro and in vivo studies demonstrating that triphenylphosphonium derivatives of BE and BA, substituted at the C-2 and C-30 positions, exhibit activity against these parasites (Figure 38). The data indicate that lupanoids with a triphenylphosphonium group at C-30 (**124**: IC_50_ = 0.64 μg/mL, **125**: IC_50_ = 0.76 μg/mL) possess approximately twice the cytotoxicity against schistosomiasis compared to analogues modified at the C-2 position (**126a**: IC_50_ = 1.5 μg/mL, **126b**: IC_50_ = 1.4 μg/mL) [150].

## 6. Compounds from Lupane Series as Potential Agents for Treatment Metabolic Disorders

The potential applications of lupanoids for managing hyperlipidemia and metabolic disorders, including non-insulin dependent diabetes mellitus, are frequently addressed in scientific literature [151,152,153,154,155,156,157,158,159,160,161,162,163]. BE, BA, and lupeol are present in medicinal plants with reported anti-diabetic properties such as *Aegle marmelos* and *Bacopa monnieri*. Research involving both in vivo and in vitro models has investigated the anti-diabetic effects of lupanoids. These studies report reductions in plasma glucose and glycated haemoglobin (HbA1c), along with increases in plasma insulin, muscle, and liver glycogen levels in diabetic rats [151]. According to research [152], results obtained in vitro indicated that triterpenoid extracts from birch bark may be suitable for wound healing in the context of diabetes. Administration of BA to obese Swiss rats at a dose of 50 mg/kg for 15 weeks, combined with a high-fat diet, resulted in decreased body weight, reduced abdominal fat accumulation, lower blood glucose, plasma triglycerides (TG), and total cholesterol levels compared to the untreated group [153]. BA demonstrated cardiovascular protective effects in both in vivo and in vitro models. For example, in an in vitro study, incubation with this acid (1 μM for 24 h) significantly inhibited the proliferation of human aortic smooth muscle cells induced by high glucose concentrations (25 μM). Additionally, BA was found to reduce hydrogen peroxide production resulting from elevated glucose levels, which subsequently decreased the formation of reactive oxygen species and helped prevent vascular endothelial injury as well as the onset of diabetic cardiovascular complications [154]. The heightened expression of cell cycle regulatory proteins, including cyclins and cyclin-dependent kinases, induced by high glucose conditions, was significantly diminished when co-incubated with BA. Furthermore, BA activated endothelial nitric oxide synthase (eNOS), thereby increasing nitric oxide production and promoting vasoprotection [155].

**Figure 39:**
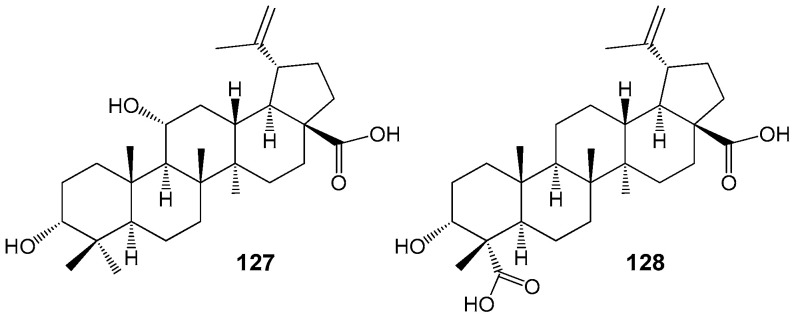
PPARγ antagonists from lupane series.

Several recent studies have demonstrated that BA and structurally related lupane compounds, including impressic acid **127** and acankoreagenin (3α-hydroxy-lup-20(29)-ene-23,28-dioic acid) **128** (Figure 39), isolated from *Schefera* plant species, are capable of forming stable complexes with peroxisome proliferator-activated receptor gamma (PPARγ) [156,157]. In traditional Chinese medicine, extracts from various *Schefera* or *Acanthopanax* species are utilized in the management of diabetes, dyslipidemia, hypertension, and other conditions, with BA regarded as a promising natural agent for the treatment of metabolic disorders [158]. Notably, this compound has been shown to modulate adipogenesis by reducing both gene and PPAR-gamma protein expression, as well as regulating co-factor specificity [158].

It was shown that acankoreagenin exhibited anti-diabetic activity, associated with the inhibition of several enzymes involved in type 2 diabetes mellitus, including α-glucosidase, protein tyrosine phosphatase 1B (PTP1B), and α-amylase (IC_50_ = 13.01, 16.39, and 30.81 μM, respectively). The compound was found to stimulate insulin release in RIN-m5F pancreatic islet cells in vitro and demonstrated a protective effect on β-cells, possibly related to the activation of the I-κBα signaling pathway, resulting in decreased expression of the transcription factor NFκB [159].

Docking analysis suggests that the orientation of the 3-hydroxy group in the molecules of BA, acankoreagenin, and impressic acid does not play a major role in the PPAR-gamma protein-lupane interaction. This may explain why other 3-epi-BA analogs, such as 3-epibetulinic acid, 3-epibetulin, and 3-epilupeol, also exhibit bioactivity [156].

BA and related compounds **127** and **128** possess a broad range of biological activity through their effects on enzymes, receptors, and transcription factors associated with type 2 diabetes. These characteristics make them potential candidates for use in the development of anti-diabetic agents.

BE has been shown to inhibit the maturation of sterol regulatory element-binding proteins (SREBPs), resulting in approximately a 40% downregulation of SREBP-2 gene expression as well as other genes associated with cholesterol, fatty acid, and triglyceride synthesis. This effect leads to decreased cellular concentrations of cholesterol and endogenous lipids. In related research, daily administration of BE at a dose of 30 mg/kg significantly improved glucose tolerance and insulin resistance in C57BL/6J mice fed a Western-type diet, as well as in those receiving a high-fat diet. Additionally, an investigation into the efficacy of BE in preventing diet-induced obesity utilized lovastatin, an HMG-CoA reductase inhibitor, as a reference compound. Both agents were administered at 30 mg/kg per day, and findings indicated that BE and lovastatin similarly reduced diet-induced obesity but via distinct mechanisms. Lovastatin appeared to reduce lipid absorption or enhance lipid excretion, whereas the anti-obesity effects of BE were attributed to increased energy expenditure. Comparable outcomes were observed in high-fat diet-fed mice [160].

Both BE and BA exhibit antioxidant properties and function as inhibitors of cytokine production, as well as TGF-β and NFκB/IκB signaling pathways [154,161]. However, in contrast to BA, BE does not have a significant effect on eNOS expression [155].

Lupeol has been identified as an antioxidant. Research indicates that it promotes the activity of superoxide dismutases (SOD), catalase (CAT), glutathione S-transferase (GST), and glutathione peroxidase (GPX). Studies have demonstrated antidiabetic and antioxidant effects of lupeol in a streptozotocin (STZ)-induced diabetes model using Wistar rats. Following lupeol treatment, reductions in HbA1c, serum glucose, and NO levels were observed, along with an increase in serum insulin levels. Additionally, administration of lupeol was associated with improvements in pancreatic antioxidants such as SOD and CAT, increased levels of reduced glutathione, GST, and GPX, and decreased reactive oxygen species induced by thiobarbituric acid. These findings suggest potential for lupeol to stimulate pancreatic regeneration, possibly through enhanced protein synthesis and antioxidant activity [162].

Although lupanoids have been experimentally validated to possess anti-diabetic properties, their efficacy remains notably lower than that of contemporary pharmaceuticals with similar indications. Nevertheless, the multifaceted actions of pentacyclic triterpenes on diabetes and its associated complications provide a rationale for developing hybrid compounds based on these molecules, aiming to create potent therapeutic agents capable of targeting various pathogenetically related aspects of the disease.

**Figure 40:**
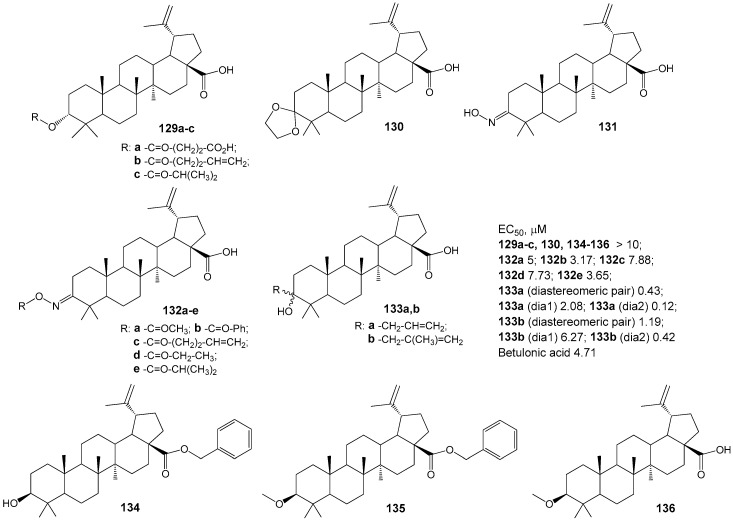
Agonists of TGR5 from BA derivatives.

The authors of the study [163] synthesized a diverse set of BA derivatives **129**–**136** with substitutions at positions C-3, C-28, and C-30 (Figure 40) and assessed their ability to modulate the G protein-coupled receptor TGR5. Activation of these receptors by natural agonists (bile acids) has been shown to influence energy homeostasis, notably enhancing energy expenditure in adipose tissue and thereby contributing to the prevention of obesity, dyslipidemia, and insulin resistance [164]. The findings revealed that compound **133a** (in its diastereomeric form) demonstrated the highest activity and selectivity as a TGR5 receptor agonist.

Clarifying the relationship between the structure of synthesized compounds and their function as TGR5 agonists has enabled the identification of critical fragments within the bile acid (BA) scaffold—specifically, the C-3 hydroxyl and free carboxylic groups—that facilitate effective biotarget binding. The study’s authors [163] further emphasize the significance of the *beta*-orientation of the 19-CH_3_ group in enhancing the affinity of BA derivatives for the receptor’s hydrophobic pocket and supporting selectivity.

**Figure 41:**
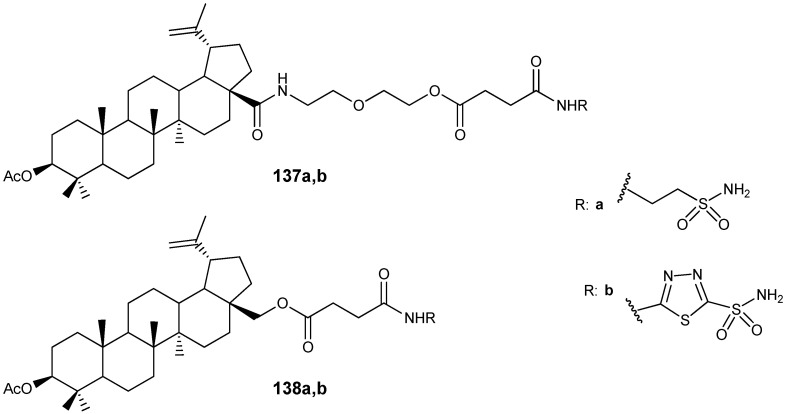
Inhibitors of carbonic anhydrase II.

It was shown above that sulfonamide conjugates of lupanoids, in addition to manifesting antitumor properties, act as inhibitors of carbonic anhydrase IX [40]. This family of enzymes, which ensures the reversible hydration of CO_2_, resulting in the formation of HCO_3_- ions, performs important functions in many biological processes, such as respiration, the maintenance of the acid-base balance, bone resorption, the formation of intraocular and cerebrospinal fluid, and others. Therefore, enzymes of this group have recently been considered not only as targets in the design of anti-glaucoma agents, but also for the correction of neuropathies, epilepsy, and edema associated with anti-amyloid therapy with monoclonal antibodies in patients with Alzheimer’s disease. Recently Denner T.-C. et al. synthesized a series of BA and BE hybrids in which the sulfonamide fragment is linked to the lupane platform via a spacer (Figure 41) and showed that compounds **137b** and **138b** act as potent competitive inhibitors of carbonic anhydrase II (Ki = 1.27 and 0.20 µM, respectively). The authors of this study attribute the greater activity of compound **137b** compared to its analogue **138b** to the longer spacer between the lupane skeleton and the sulfamide moiety in its molecule. Using molecular modelling calculations, they showed that with a longer spacer in the molecule, the sulfamide fragment more easily penetrates the active site of the enzyme [165].

## 7. Triterpenoid Derivatives from Lupane Series in Materials Science

The modification of natural chiral compounds for materials science plays a critical role in advancing optoelectronic technologies, particularly in the development of compact devices such as color electronic paper and books, trademarks, advertising displays, information boards, and screens for digital navigators. These advancements contribute to the broader application of displays utilizing chiral-nematic (cholesteric) liquid crystal (LC) mixtures, which selectively reflect circularly polarized light within the visible spectrum. In comparison to alternative LC display technologies, these devices offer exceptionally low energy consumption, owing to the absence of energy-intensive backlighting and the presence of a memory effect. Additionally, they feature enhanced contrast and a wide viewing angle [166,167].

Selective reflection of circularly polarized light in a chiral-nematic liquid crystal (LC) mixture arises from the induction of a spiral supramolecular architecture by a chiral dopant (CD). To achieve this effect within the visible spectrum, the cholesteric helix must possess a sufficiently short pitch (P ~0.3–0.6 μm). The quantitative parameter describing the ability to induce such a helical structure is the twisting power (β) of the CD. Nevertheless, incorporating a CD into the nematic solvent can influence key properties, including the temperature range of the mesophase, threshold voltages for control, viscosity, and switching speed. Accordingly, CDs should exhibit high twisting power to facilitate their use at low concentrations in nematic solvents. Additional critical characteristics of these LC systems include a minimal temperature dependence of the reflected light’s maximum wavelength (dλ_max_/dT ≈ 0 within 0–50 °C), along with robust photo- and phase stability. These attributes are determined by specific features of the molecular structure of the CD [166,167,168,169].

To date, the most frequently studied chiral dopants (CDs) exhibiting strong twisting abilities in liquid crystal (LC) mixtures include dioxolane derivatives (TADDOL), symmetrically substituted dianhydro-D-hexitols, and axially chiral binaphthyl derivatives (BINOL). These compounds are characterized by the presence of a chiral backbone and multiple promesogenic substituents with extended π-electron systems, which contribute to their compatibility with LC matrices. Each structural type presents specific limitations: BINOLs exhibit challenges in synthesis and display low solubility within LC matrices, while TADDOL and dianhydro-D-hexitols show temperature dependence of λ_max_ reflected light. The introduction of additional components can address some of these issues but may also adversely affect the material properties [167,170,171].

**Figure 42:**
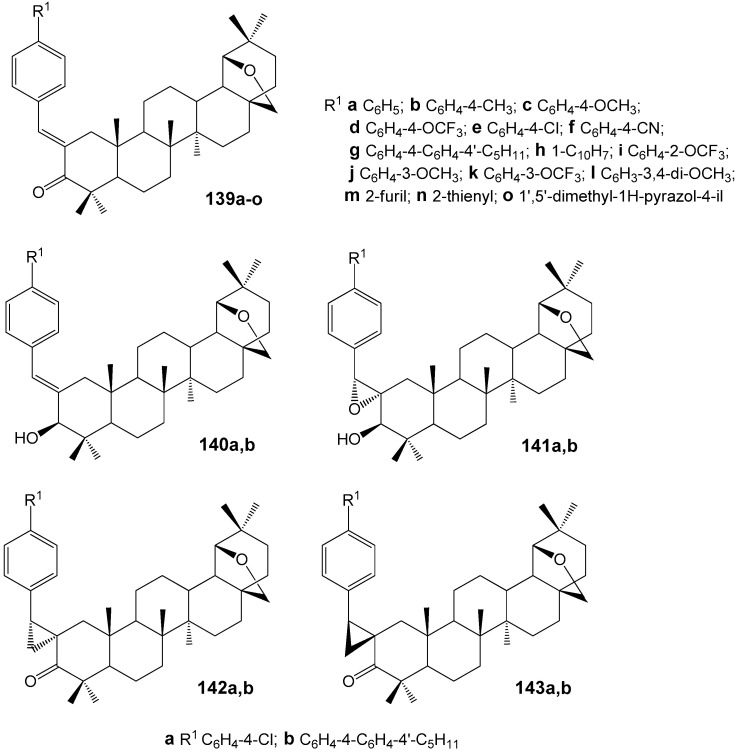
2-Arylmethyliden derivatives of the lupane series.

The development of more effective dopants can be addressed by exploring new structural types, particularly through the synthesis of enantiomerically pure triterpenoids with more complex scaffolds than those previously tested. Pentacyclic triterpenoids of the lupane series as chiral carriers for optically active components of liquid crystal materials have only recently been discussed in scientific and informational literature. Studies [172,173,174] have examined the synthesis of allobetulon **139** and allobetulin **140** 2-ylidene derivatives (Figure 42), their conversion into compounds containing oxirane **141** and cyclopropane **142**, **143** groups, and their twisting power in the common nematic solvent 4-cyano-4′-pentylbiphenyl (5CB). Compounds **140b**–**143b**, which include developed promesogenic fragments at the C-2 atom, demonstrated a higher ability to induce helical supramolecular structures (Table 2) compared to their synthetic precursor, the α,β-unsaturated ketone **139g**.

**Figure 43:**
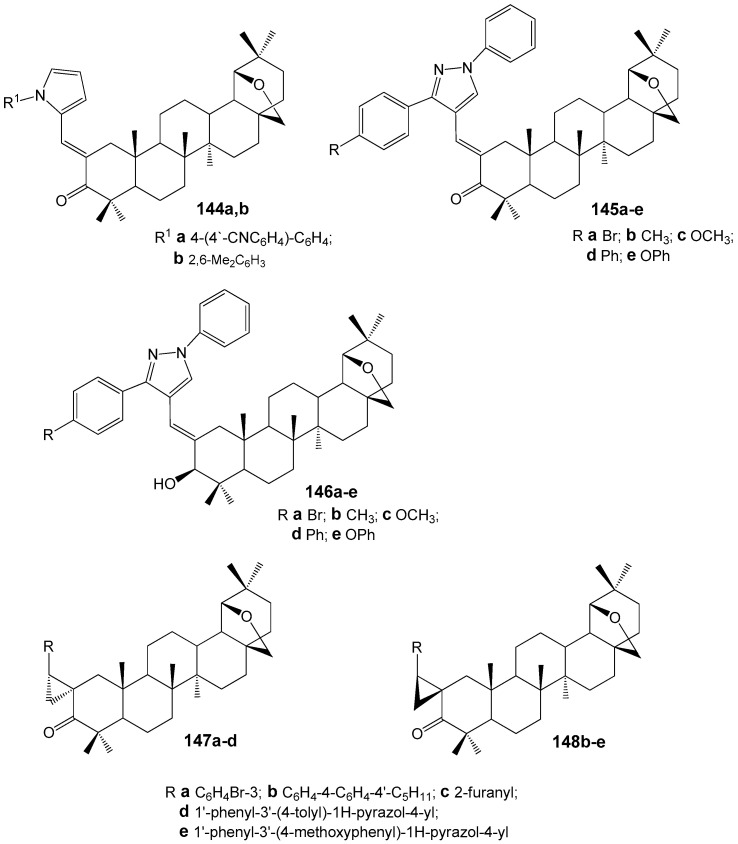
2-Hetarylmethylidene and 2-cyclopropyl derivatives of the lupane series.

For the 2-hetarylmethylidene derivatives **144**–**146** (Figure 43), which were synthesized by condensing allobetulone with pyrrole and pyrazole aldehydes, unsaturated ketones **144a** and **145a**–**e**, except for compound **144b** with the shortest conjugation chain, exhibited higher induction of the cholesteric mesophase in the nematic solvent 5CB (Table 3). The reduction products of ketones **145**, namely allyl alcohols **146a**–**e**, showed lower twisting power compared to their synthetic precursors [173].

Based on ^1^H NMR data, X-ray diffraction studies, and quantum chemical calculations, researchers [175,176] determined that the twisting power of CDs is affected by the size and geometry of the π-electron systems of substituents at the C-2 atom of the lupane core, as well as the inclination angle of the promesogenic fragment’s axis relative to the triterpenoid platform. For example, in the 4′-pentyl-1,1′-biphenyl substituent, a common promesogenic fragment, the aryl rings are oriented linearly, whereas in N-aryl-substituted pyrazole moieties, the axes of aromatic rings at the N and C atoms are positioned at an angle. The incorporation of these fragments into the CD structure alters the overall molecular shape, which subsequently influences the twisting power of these compounds. The presence of spiro-cyclopropane and spiro-oxirane substituents at the C-2 atom also changes the spatial relationship between the promesogenic fragment and the lupane platform.

Thus, the bulkier the substituent with a developed π-electron system is at the C-2 position, the higher the twisting force. A comparison of the |β| values with the geometric data on the structure of the CDs obtained from X-ray diffraction studies indicates that a smaller dihedral angle between such a substituent and the plane of the lupane core correlates with higher |β| values.

CDs **141b**, **142b** (see Figure 42), and **148d**,**e** (see Figure 43) when mixed with commercial nematics (C7, CL037, CL038, and LCM-1847—characterized by exceptionally low threshold control voltages and a broad mesophase temperature range from <–10 °C to >+60 °C) showed selective light reflection spanning from blue to red at low concentrations (3–5 wt.%), along with high solubility within liquid crystals and notable phase stability in the mixtures. These properties confirm their potential for practical applications.

**Figure 44:**
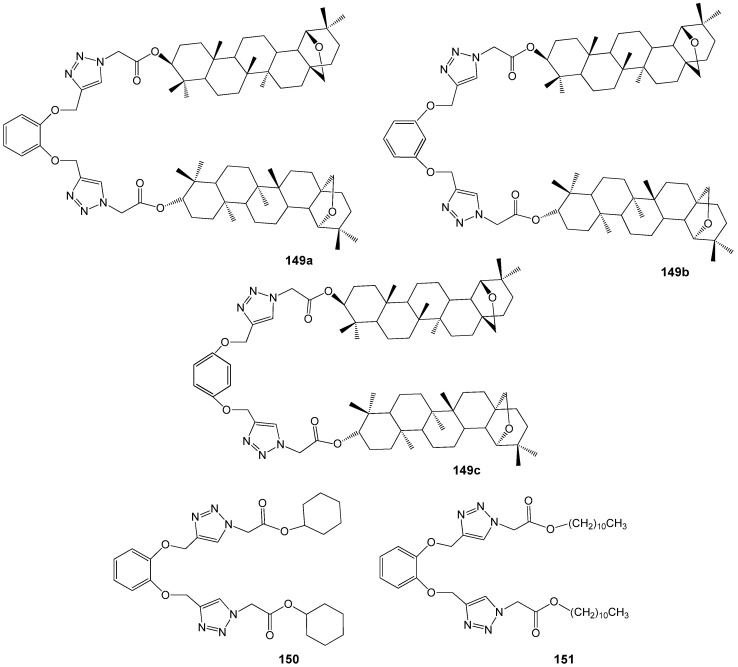
Allobetuline appended 1,2,3-triazole-based gelators and model compounds.

Low-molecular-weight organogelators featuring triterpenoid or steroid scaffolds are of considerable interest due to their nano-sized molecular platforms and broad potential applications, such as pollutant removal and chemical sensing [177,178,179,180,181,182,183,184,185,186]. However, prior to the publication of the article [186], there were no studies in the literature devoted to lupane derivatives as components of organogels. Most publications examine numerous steroid derivatives. Among pentacyclic triterpenoids, gelation properties have been observed in conjugates of arjunolic and glycyrrhetinic acids [179,180,181,182]. The influence of various structural fragments on the self-assembly processes of these compounds in certain solvents also remains insufficiently understood. The authors of most studies emphasize the predominant role of H-bonds between the gelator and the solvent, as well as π-stacking of aromatic linkers that are part of the gelling agents [177,184]. Reference [186] describes the synthesis of three novel organogelator candidates **149a**–**c** (Figure 44), where two allobetulin units are connected by an aromatic spacer through 1,2,3-triazole linkers. Additionally, two model compounds **149** and **150** designed similarly incorporate cyclohexane and undecane segments in place of lupane fragments. Their gelation properties were evaluated experimentally in twelve organic solvents, as well as computationally using molecular dynamics and quantum-chemical approaches. The study determined that the 1,2,3-triazole derivative of allobetuline **149a** with a 1,2-dihydroxybenzene spacer formed a stable gel exclusively in toluene. In contrast, the model compounds **150**, **151** did not exhibit gel formation in any of the solvents tested.

Molecular dynamics simulations conducted in six solvents (water, ethanol, cyclohexanol, acetonitrile, toluene) alongside quantum-chemical calculations demonstrate that intermolecular interactions of the tail substituents are the primary factor influencing the gelation of the synthesized compounds. In contrast, molecular unfolding within the solvent plays a less significant role, instead promoting the solubility of the gelling agent. Consequently, classical molecular dynamics modeling enables the identification of preferred molecular conformations and supports the proposal of optimal structures for potential gelators [186].

## 8. Conclusions

The need to find new, more effective antitumor, antiviral, antibacterial, anti-inflammatory, and antiparasitic drugs than those currently available is prompting researchers to turn to natural metabolites with unique properties. Lupane triterpenoids, along with other pentacyclic triterpenoids, are attractive candidates for the development of innovative drugs. In recent years, many hybrid compounds have been developed in which the lupane platform is linked to another molecule via an ester, amide, hydrazide, ureide, or carbamate linkage, often as part of a spacer moiety. Heterocyclic fused systems based on the lupane skeleton have also been obtained. Compared to natural lupanoids a significant increase in antitumor activity (compounds **7a**,**b**, and **11b**) and antiviral activity (conjugates **75**–**77**) was achieved. The combination of high cytotoxicity with the inhibition of certain enzymes, such as carbonic anhydrases IX and XII, observed in betulin-sulfonamides **34a**,**b** is an advantage, allowing them to be considered promising lead structures for further pharmacological trials. Among the substances with antiparasitic properties, the antimalarial activity of artesunic acid conjugates **110** and **113** is noteworthy. The synthesis of PEG conjugates that provide targeted delivery to tumor cells is undoubtedly a major achievement. However, despite some successes, the limiting factor for the further development of most conjugates as drug candidates, apart from their insufficient specific activity, remains their low water solubility, which has a negative impact on their bioavailability in vivo. At the same time, the high lipophilicity of lupanoids supports their use as chiral dopants in liquid crystal compositions or as components in organogels. However, this area of application of modified lupanoids remains the least studied. The present analysis of publications shows that the research of hybrid compounds based on lupane triterpenoids is an intensively developing direction with high expectations. It can be assumed that in the near future, new promising routes for the chemical transformation of these natural compounds will be found which will make it possible to obtain both highly active substances with pharmacokinetic parameters acceptable for their subsequent medical use, as well as new components and supramolecular systems of interest for materials science.

## Figures and Tables

**Table 1:** The cytotoxicity of BA and its hybrids (IC_50_, µM) against DLD-1, HT-29, HeLa cancer cell lines.

Compound	DLD-1	HT-29	HeLa
BA	70.75 ± 0.01	87.05 ± 3.89	62.65 ± 12.17
BA-levulinate	15.42 ± 1.58	33.13 ± 13.14	37.90 ± 0.01
methylated BA-levulinate	105.19 ± 10.05	68.70 ± 3.54	63.61 ± 1.55
**53**	64.20 ± 5.28	27.72 ± 9.31	40.90 ± 11.98

**Table 2:** Twisting power (β) of compounds **139e,g**, **140a,b–143a,b** in nematic solvent 5CB.

Compound	139e	140a	141a	142a	143a
|β|, (µm^−1^ mol. pats^−1^)	29.7 ± 1.4	71.4 ± 3.4	84.3 ± 3.7	57.8 ± 2.6	3.4 ± 0.2
**Compound**	**139g**	**140b**	**141b**	**142b**	**143b**
|β|, (µm^−1^ mol. pats^−1^)	10.8 ± 0.5	144.5 ± 3.1	174.9 ± 3.9	174.3 ± 0.9	122.5 ± 3.0

**Table 3:** Twisting power (β) of compounds **145–149** in nematic solvent 5CB.

Compound	144a	144b	145a	145b	145c	145d
|β|, (µm^−1^ mol. pats^−1^)	89.7 ± 2.5	6.8 ± 1.1	108.9 ± 4.3	129.9 ± 2.8	145.0 ± 1.3	158.6 ± 8.7
**Compound**	**145e**	**146a**	**146b**	**146c**	**146d**	**146e**
|β|, (µm^−1^ mol. pats^−1^)	106.4 ± 2.4	51.9 ± 1.6	52.7 ± 2.1	63.8 ± 0.3	61.7 ± 1.3	49.2 ± 2.0
**Compound**	**147a**	**147b**	**147c**	**147d**	**148b**	**148c**
|β|, (µm^−1^ mol. pats^−1^)	5.2 ± 0.4	174.7 ± 0.9	25.66 ± 0.5	107.5 ± 3.3	123.5 ± 3.0	61.8 ± 0.4
**Compound**	**148d**	**148e**				
|β|, (µm^−1^ mol. pats^−1^)	213.8 ± 1.1	230.5 ± 4.3				
